# Supplementary figures and images for: An anchor in troubled times: Trust in science before and within the COVID-19 pandemic
Source: PLoS One. 2022 Feb 9;17(2):e0262823. doi: 10.1371/journal.pone.0262823 (PMC8827432; doi:10.1371/journal.pone.0262823)

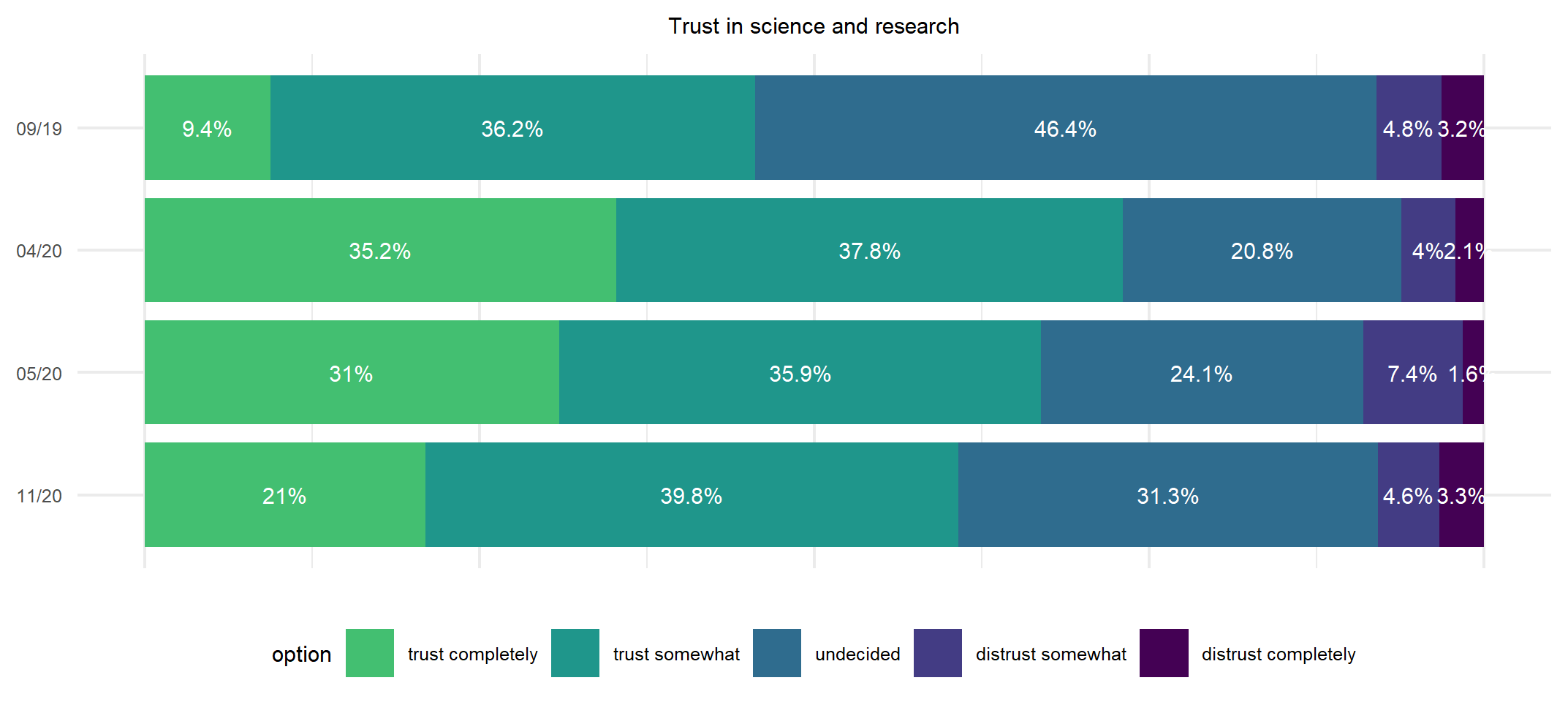

Supplement: S1 Fig — (TIFF) [file pone.0262823.s001.tiff]

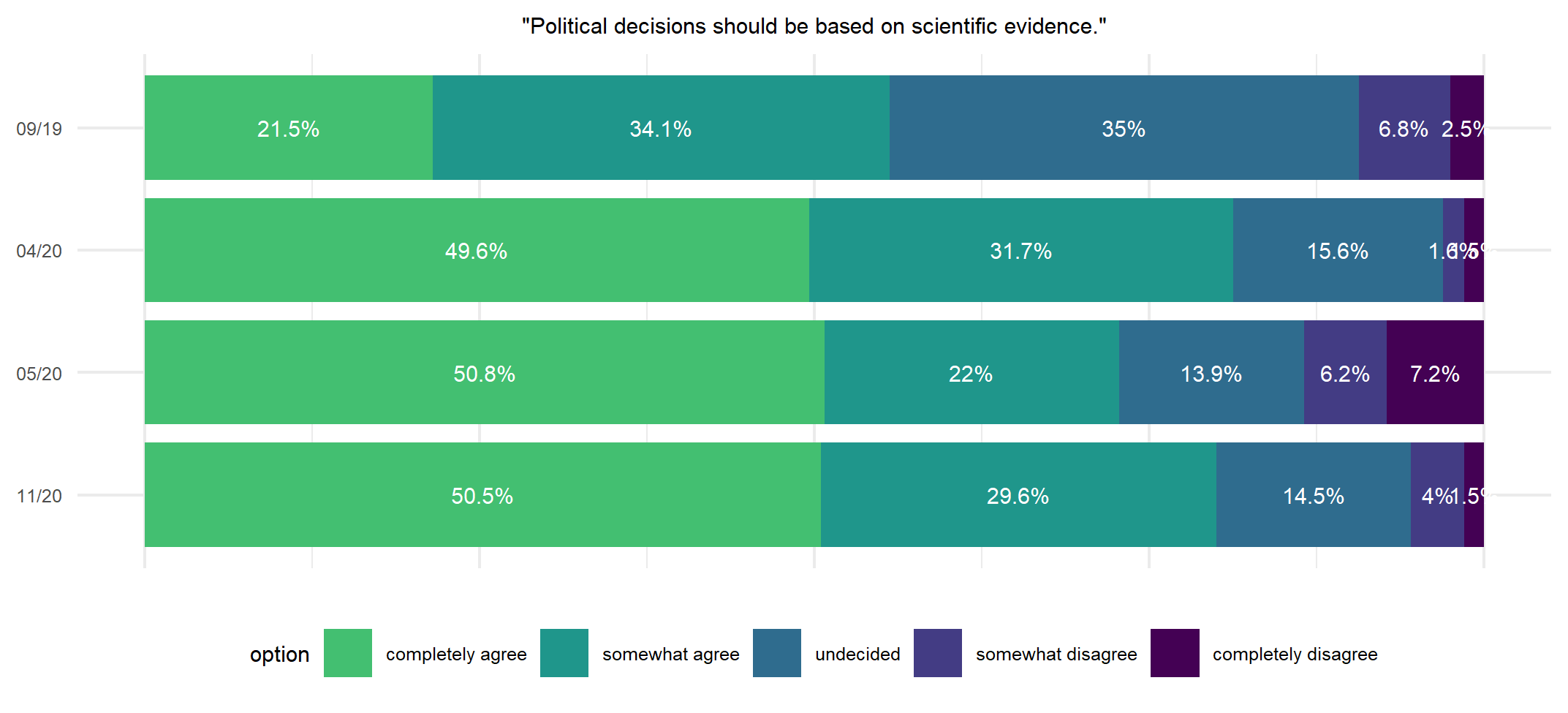

Supplement: S2 Fig — (TIFF) [file pone.0262823.s002.tiff]

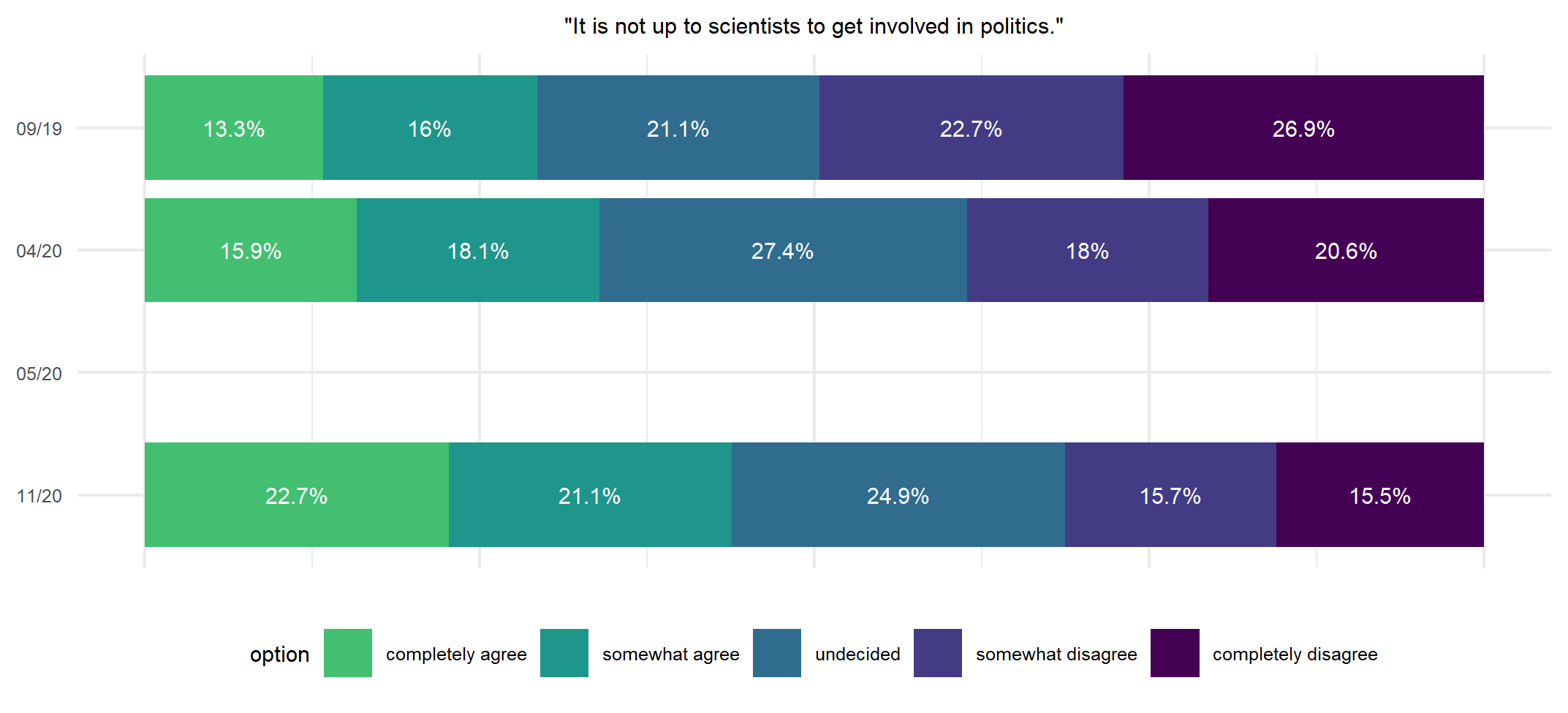

Supplement: S3 Fig — (TIFF) [file pone.0262823.s003.tiff]

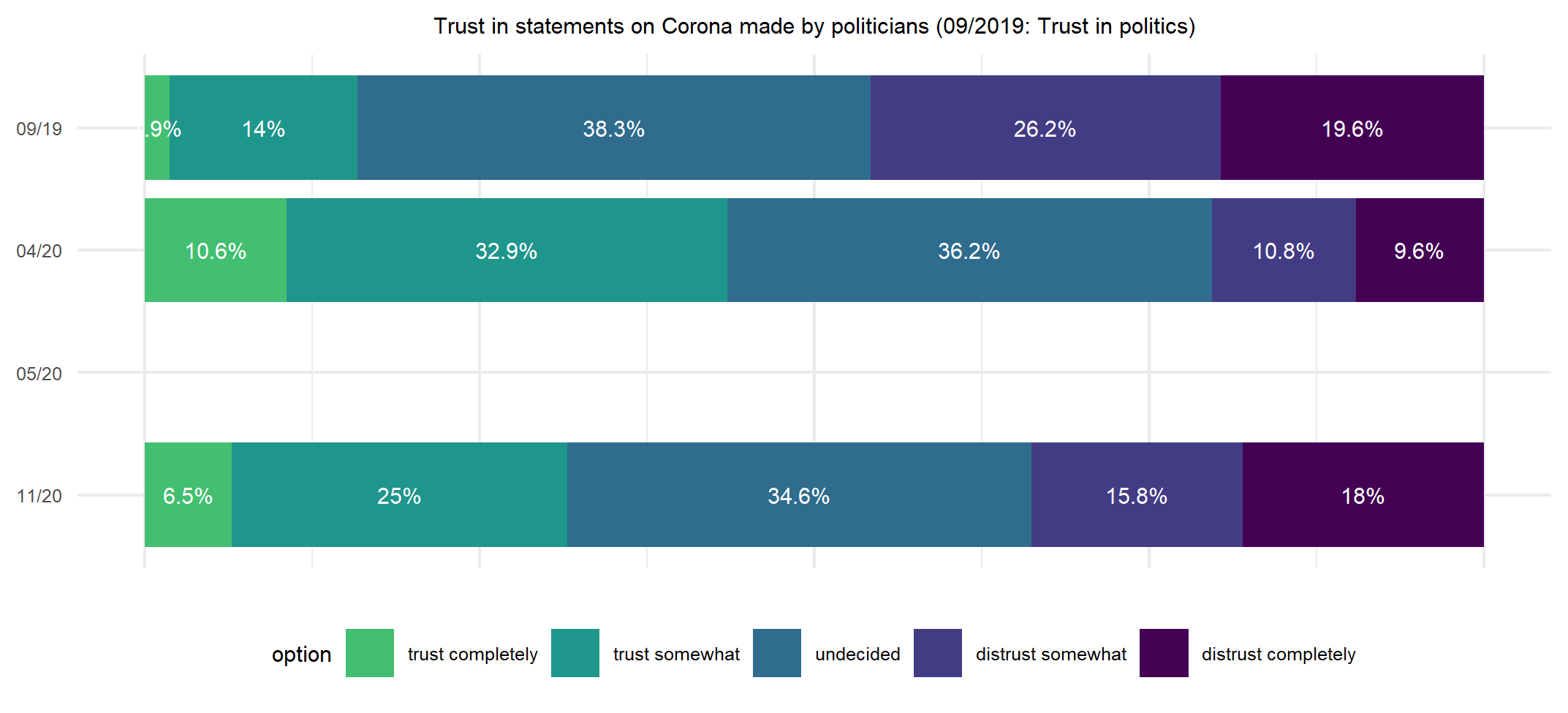

Supplement: S4 Fig — (TIFF) [file pone.0262823.s004.tiff]

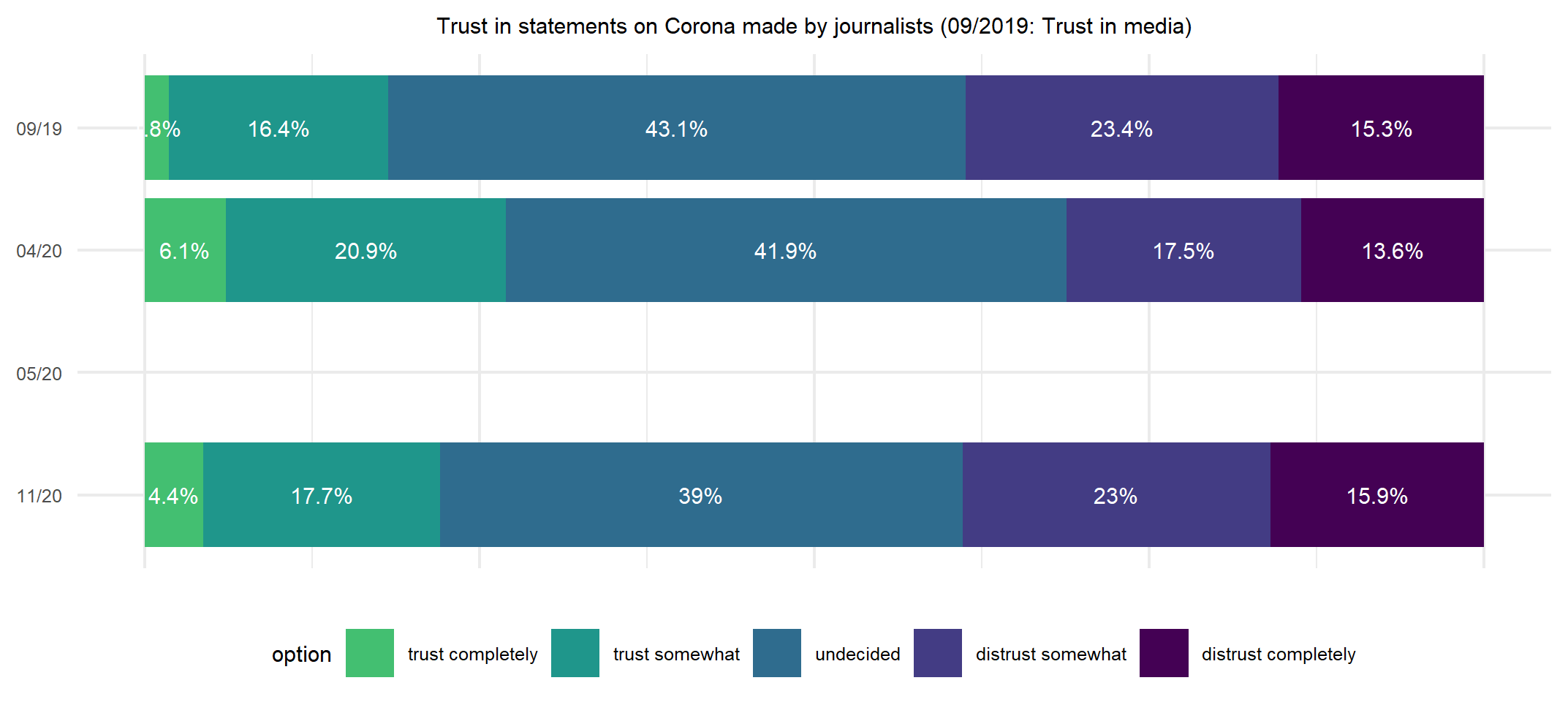

Supplement: S5 Fig — (TIFF) [file pone.0262823.s005.tiff]

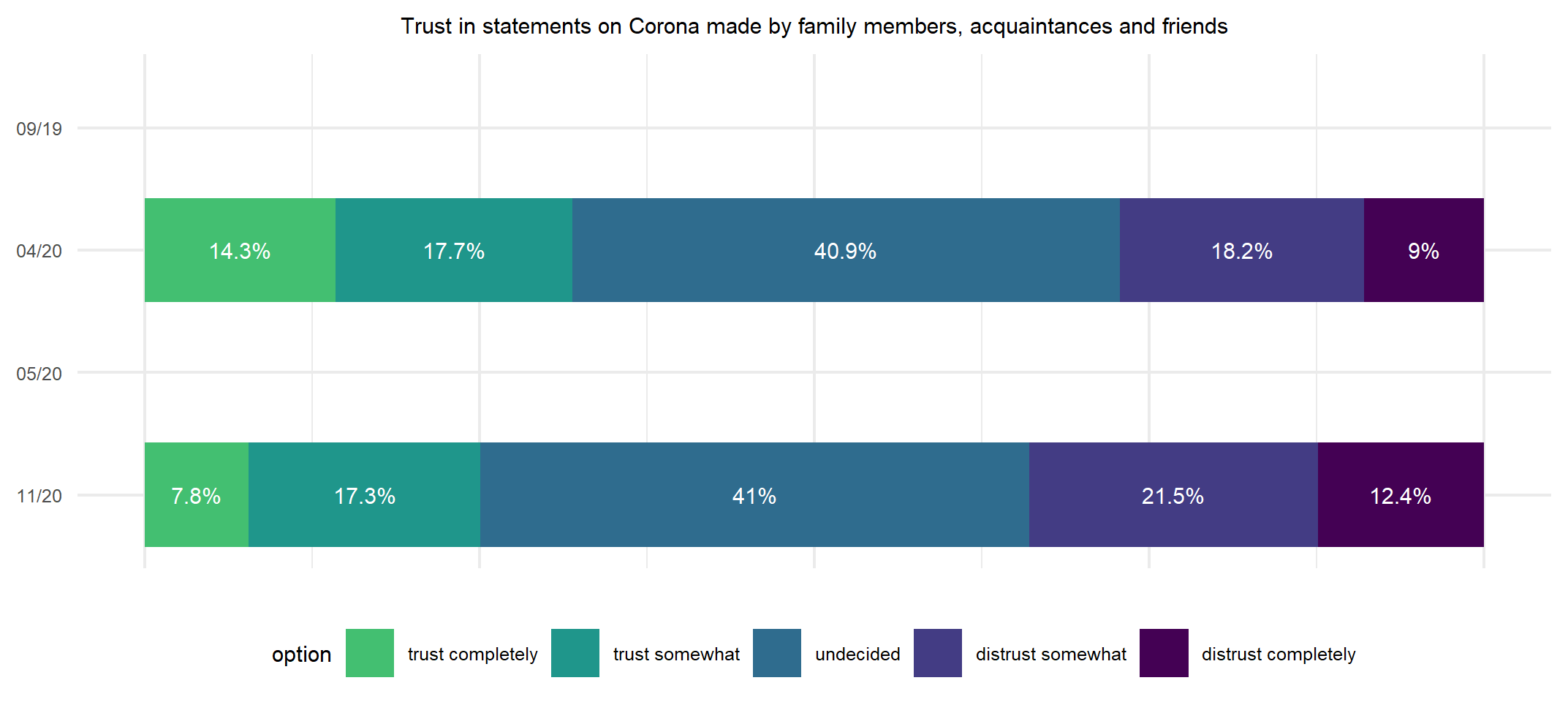

Supplement: S6 Fig — (TIFF) [file pone.0262823.s006.tiff]

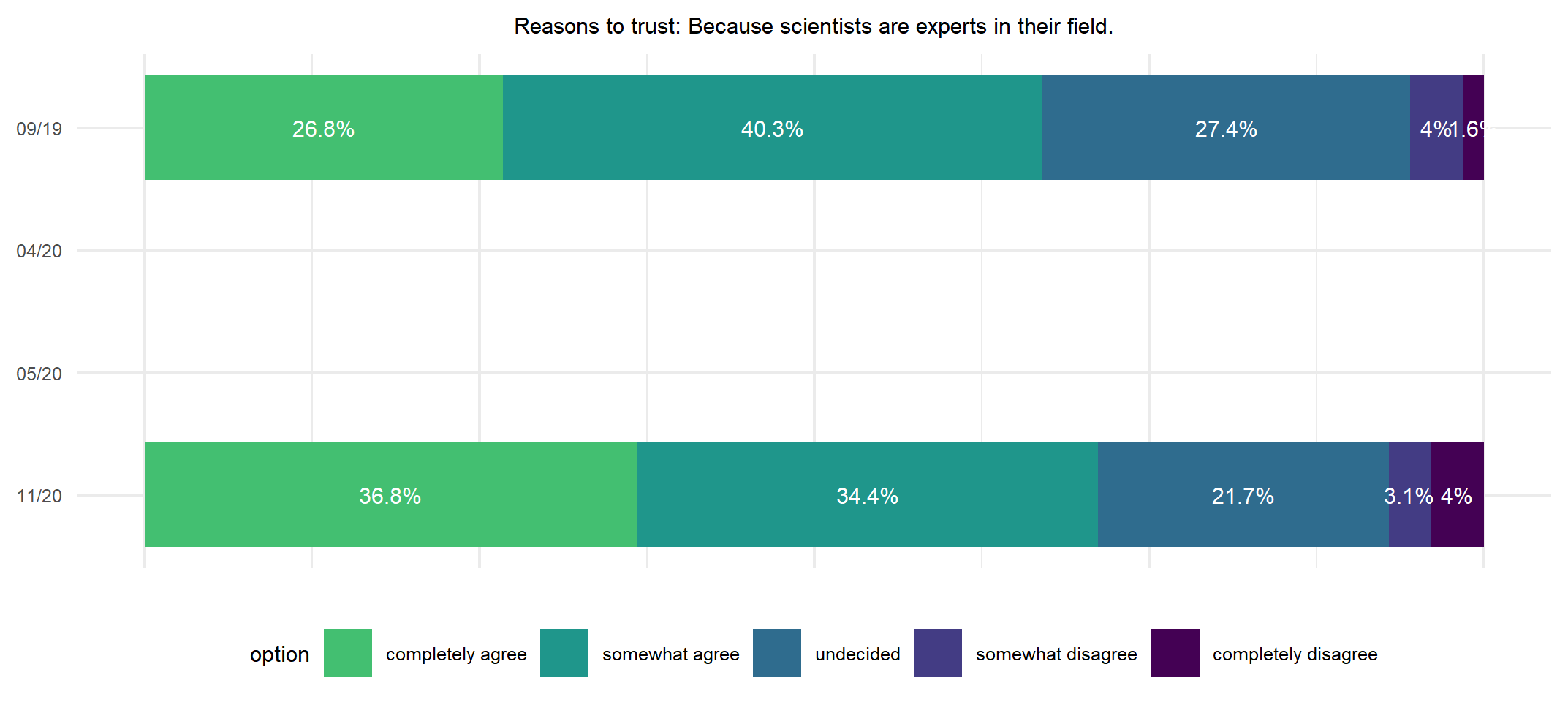

Supplement: S7 Fig — (TIFF) [file pone.0262823.s007.tiff]

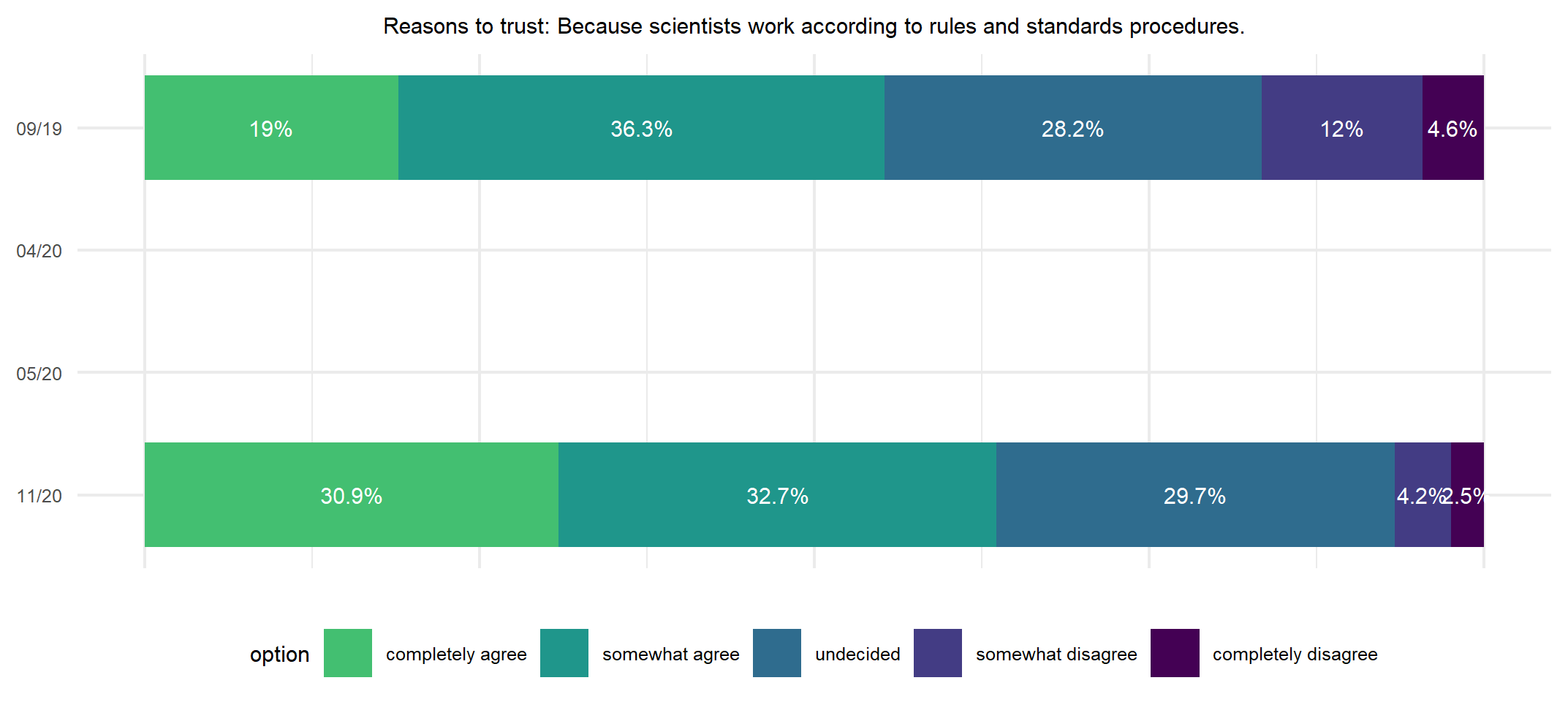

Supplement: S8 Fig — (TIFF) [file pone.0262823.s008.tiff]

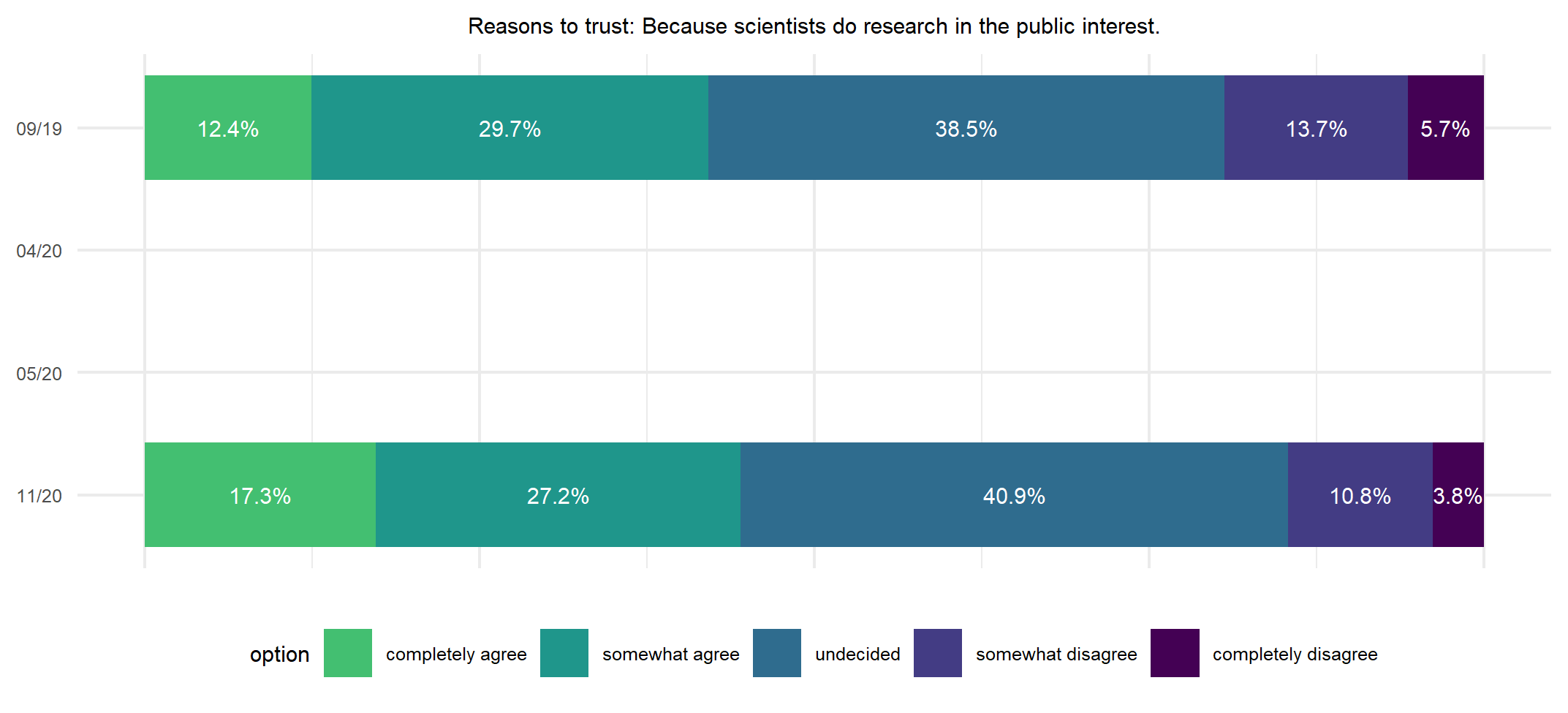

Supplement: S9 Fig — (TIFF) [file pone.0262823.s009.tiff]

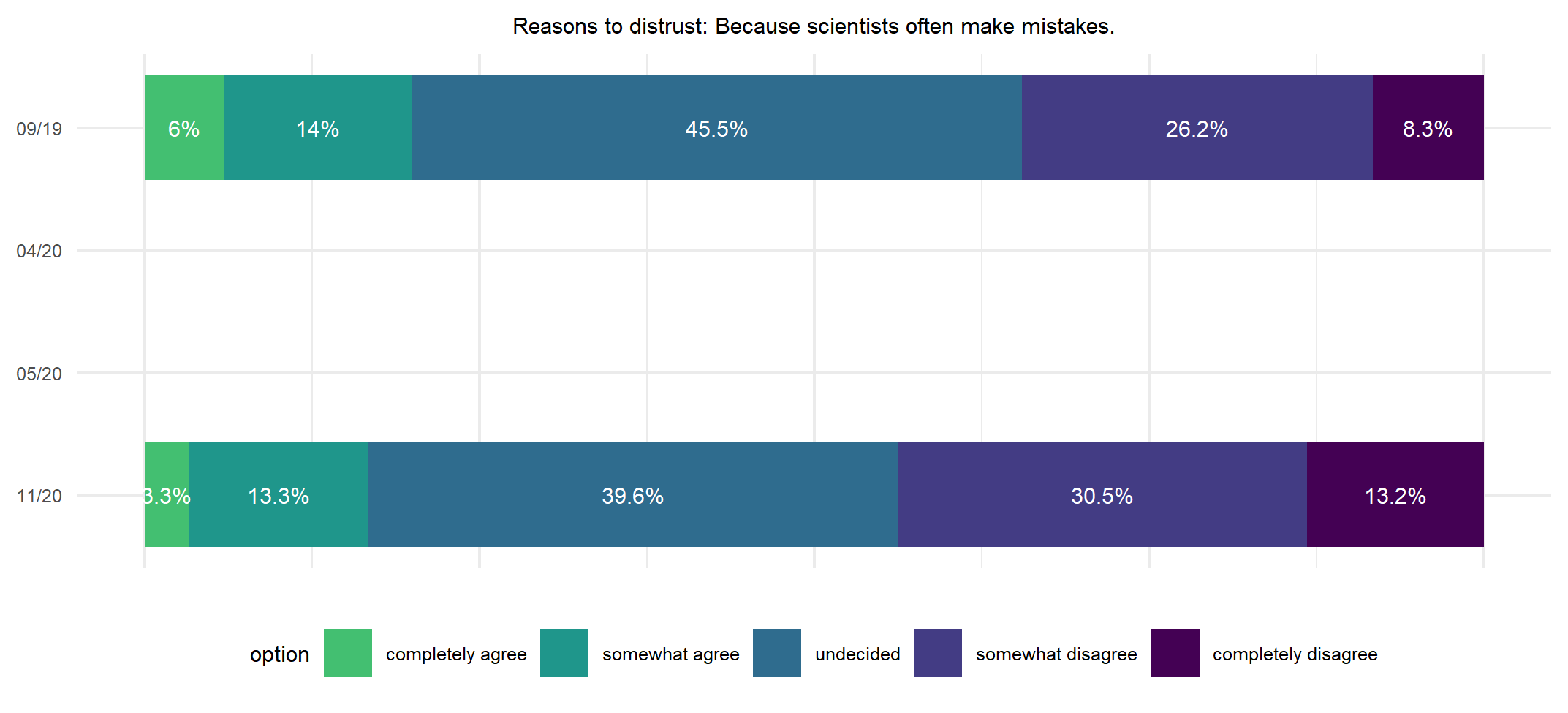

Supplement: S10 Fig — (TIFF) [file pone.0262823.s010.tiff]

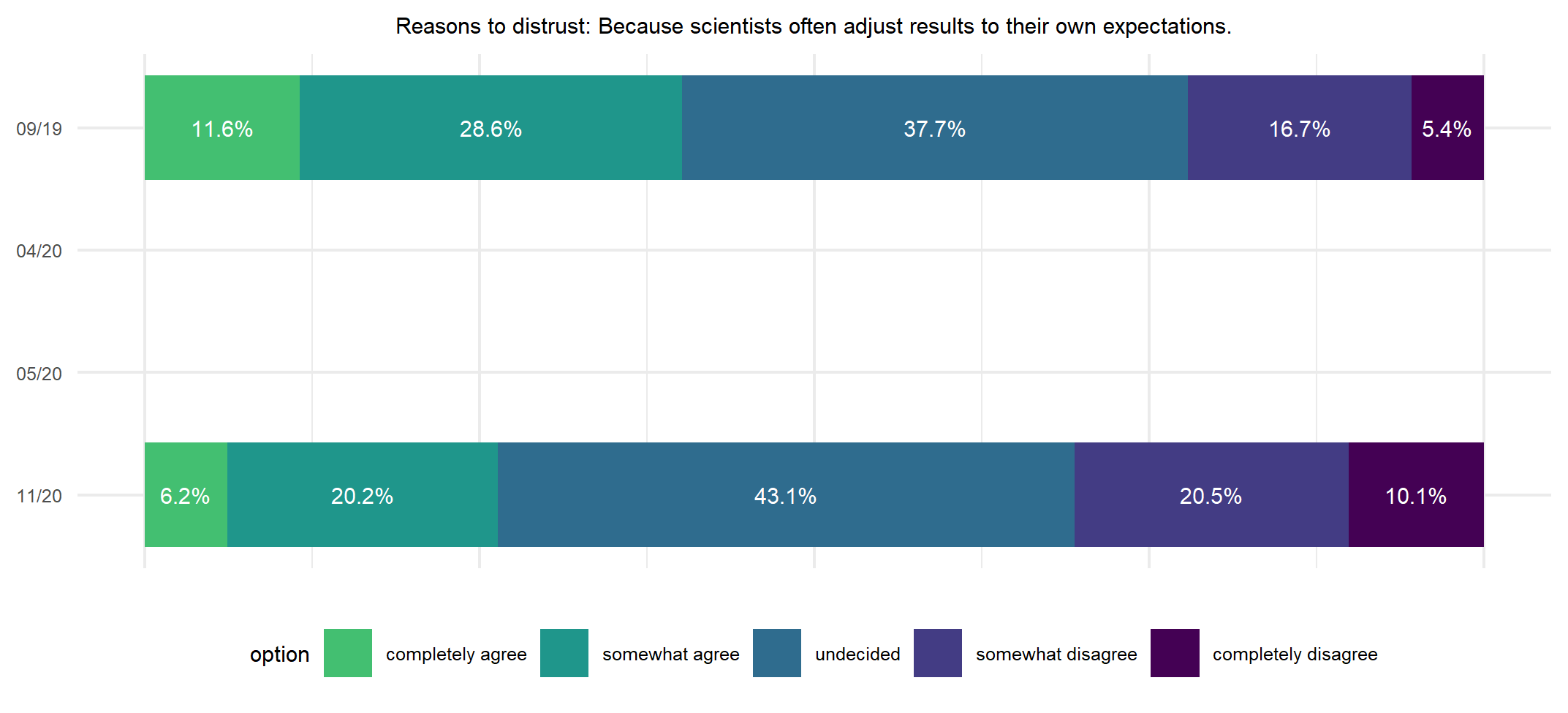

Supplement: S11 Fig — (TIFF) [file pone.0262823.s011.tiff]

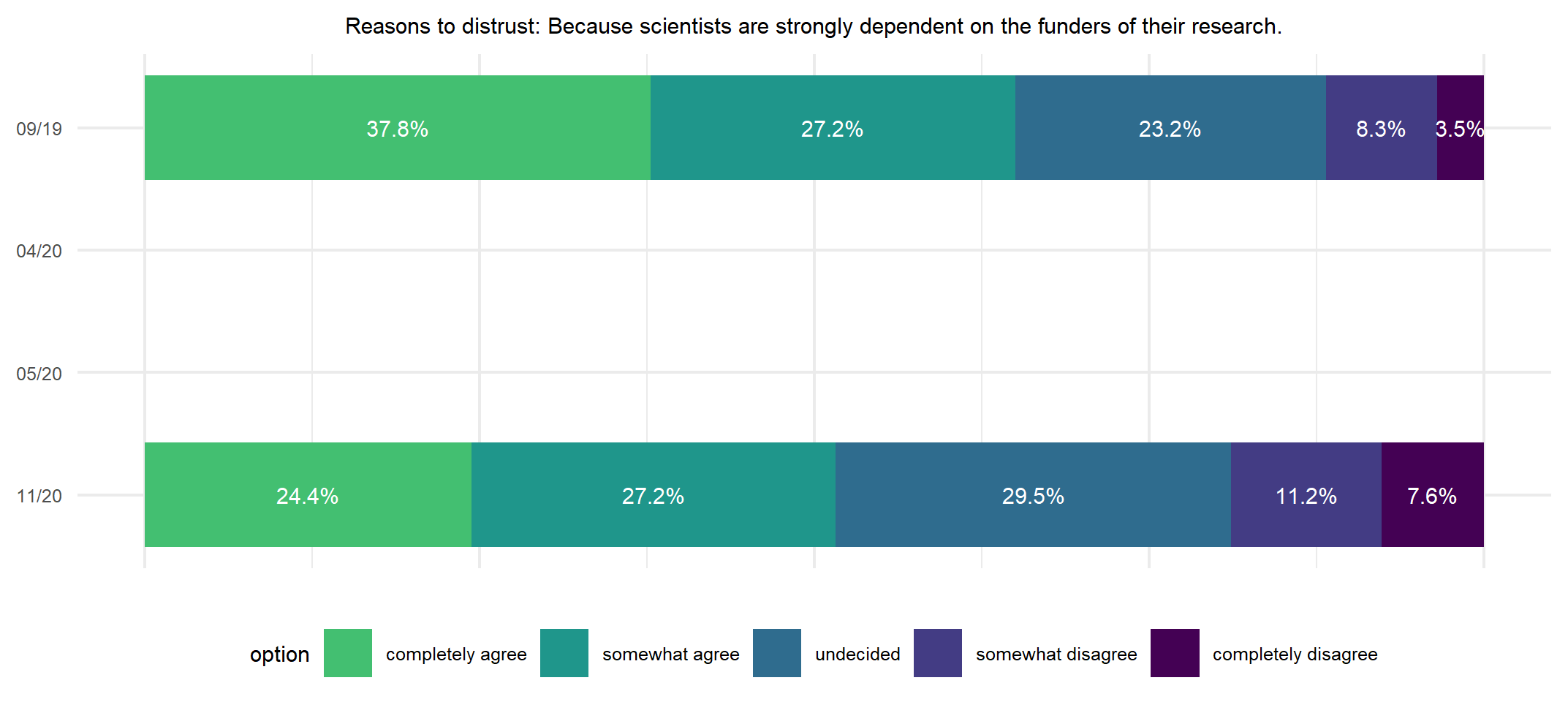

Supplement: S12 Fig — (TIFF) [file pone.0262823.s012.tiff]

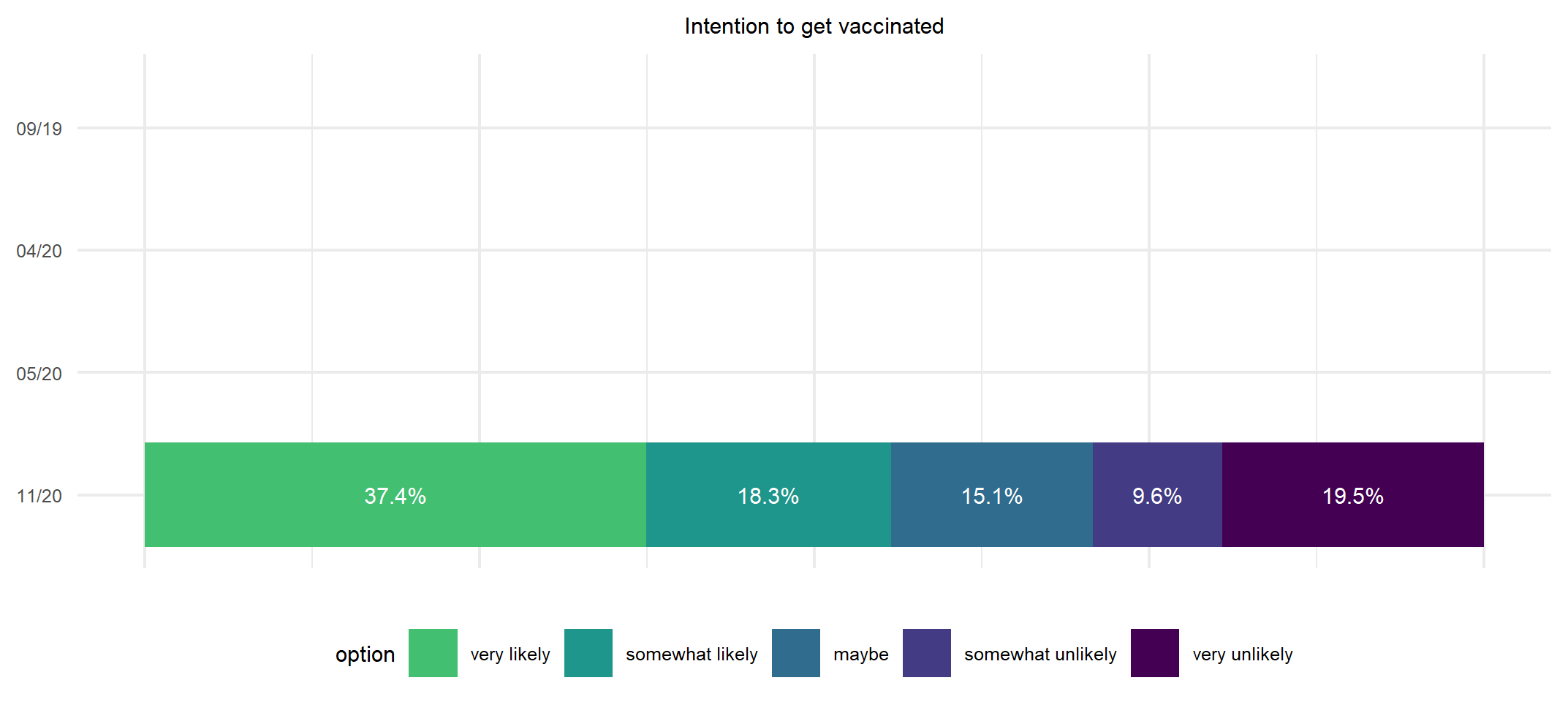

Supplement: S13 Fig — (TIFF) [file pone.0262823.s013.tiff]

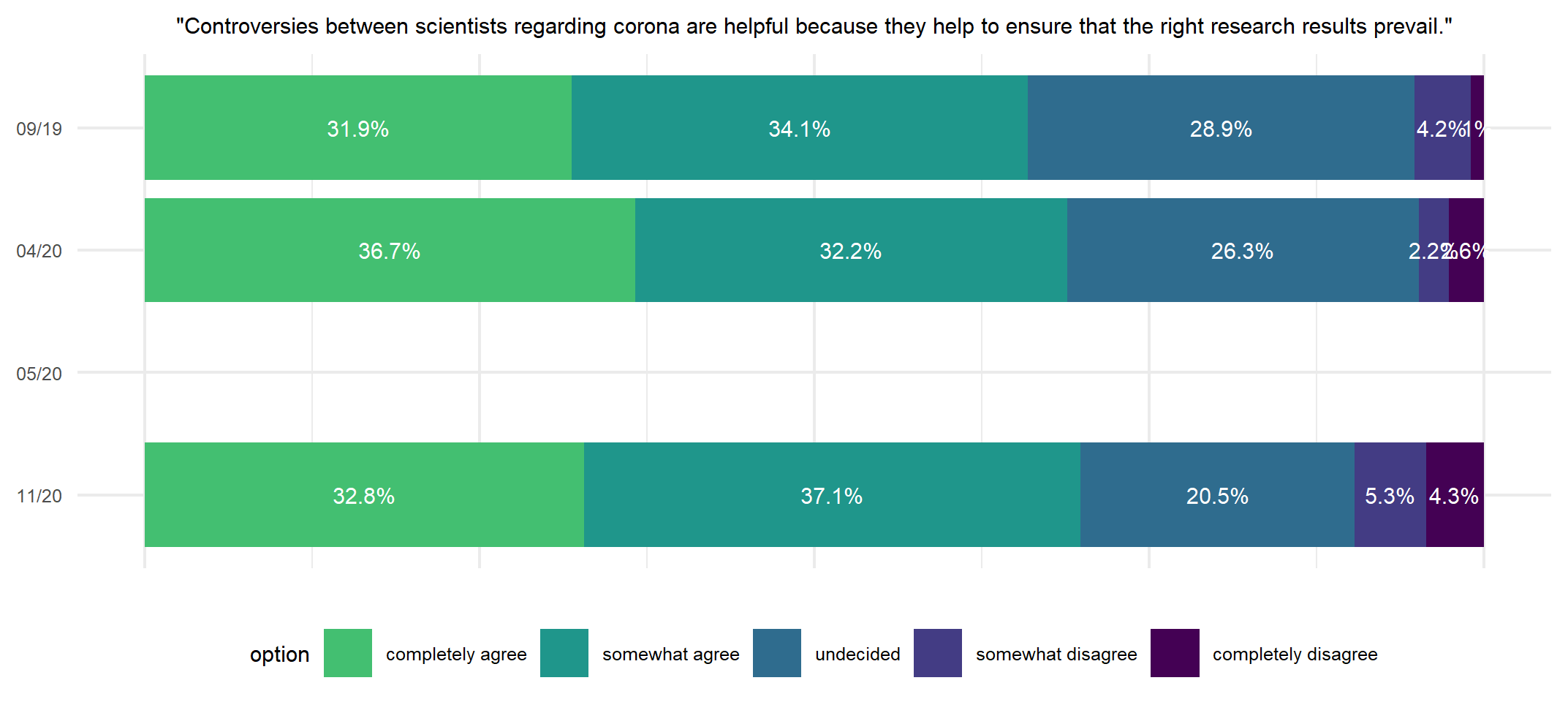

Supplement: S14 Fig — (TIFF) [file pone.0262823.s014.tiff]

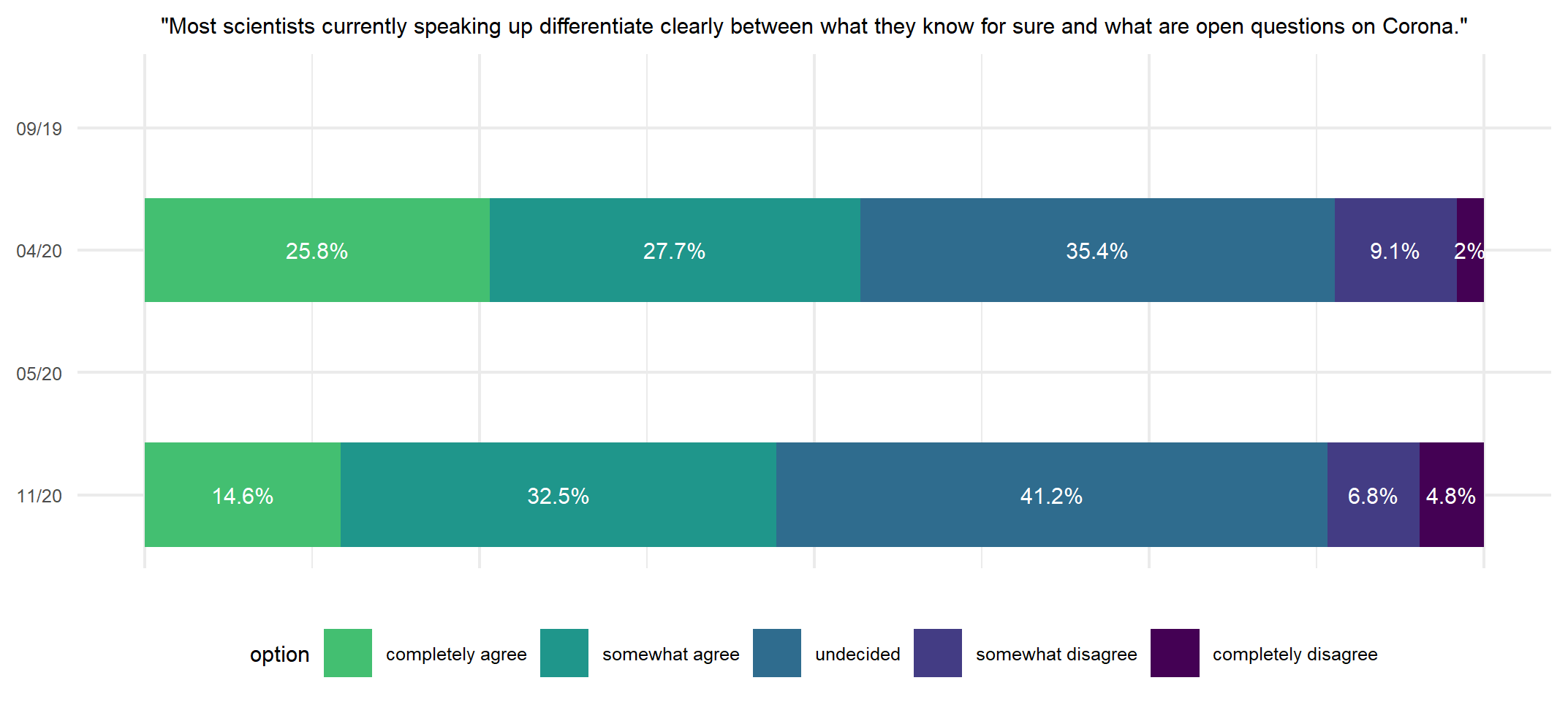

Supplement: S15 Fig — (TIFF) [file pone.0262823.s015.tiff]

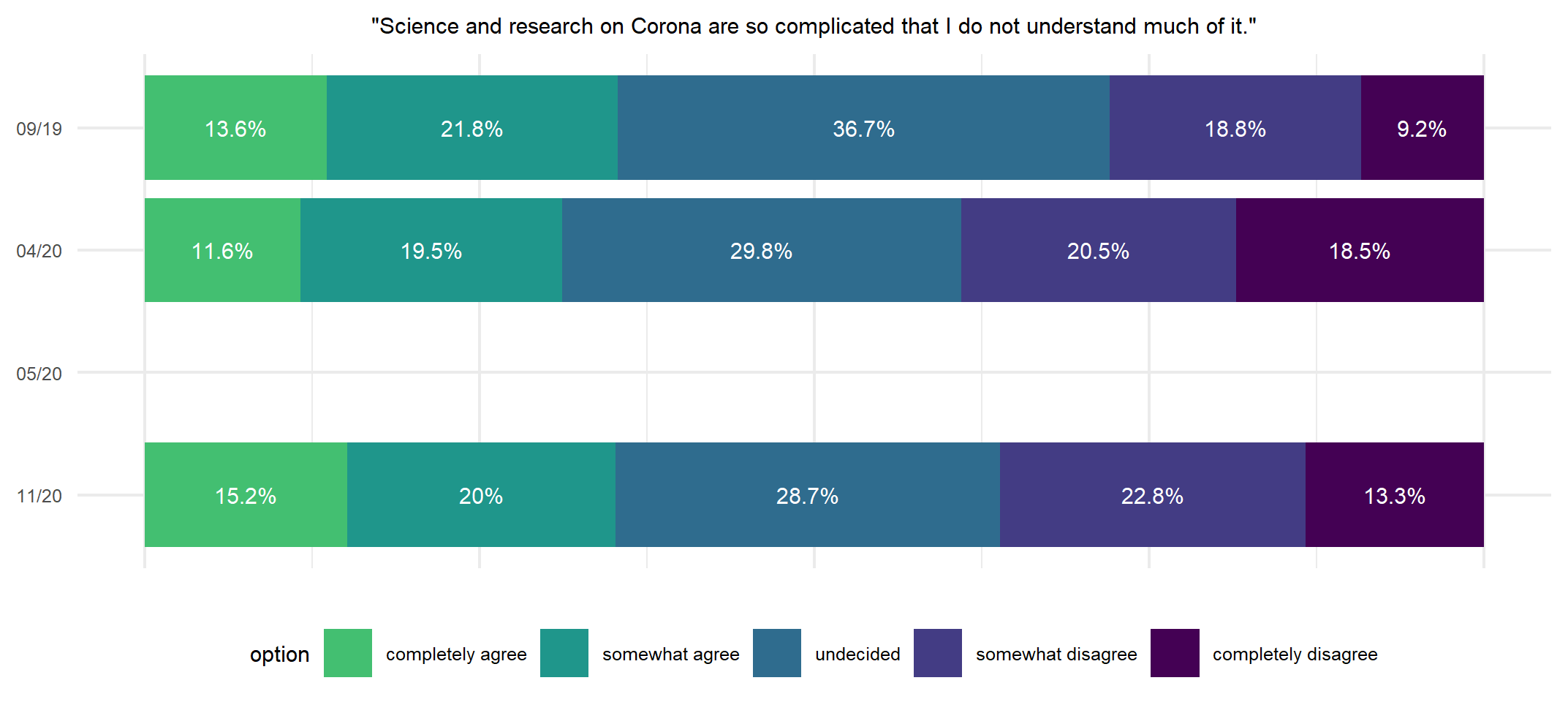

Supplement: S16 Fig — (TIFF) [file pone.0262823.s016.tiff]

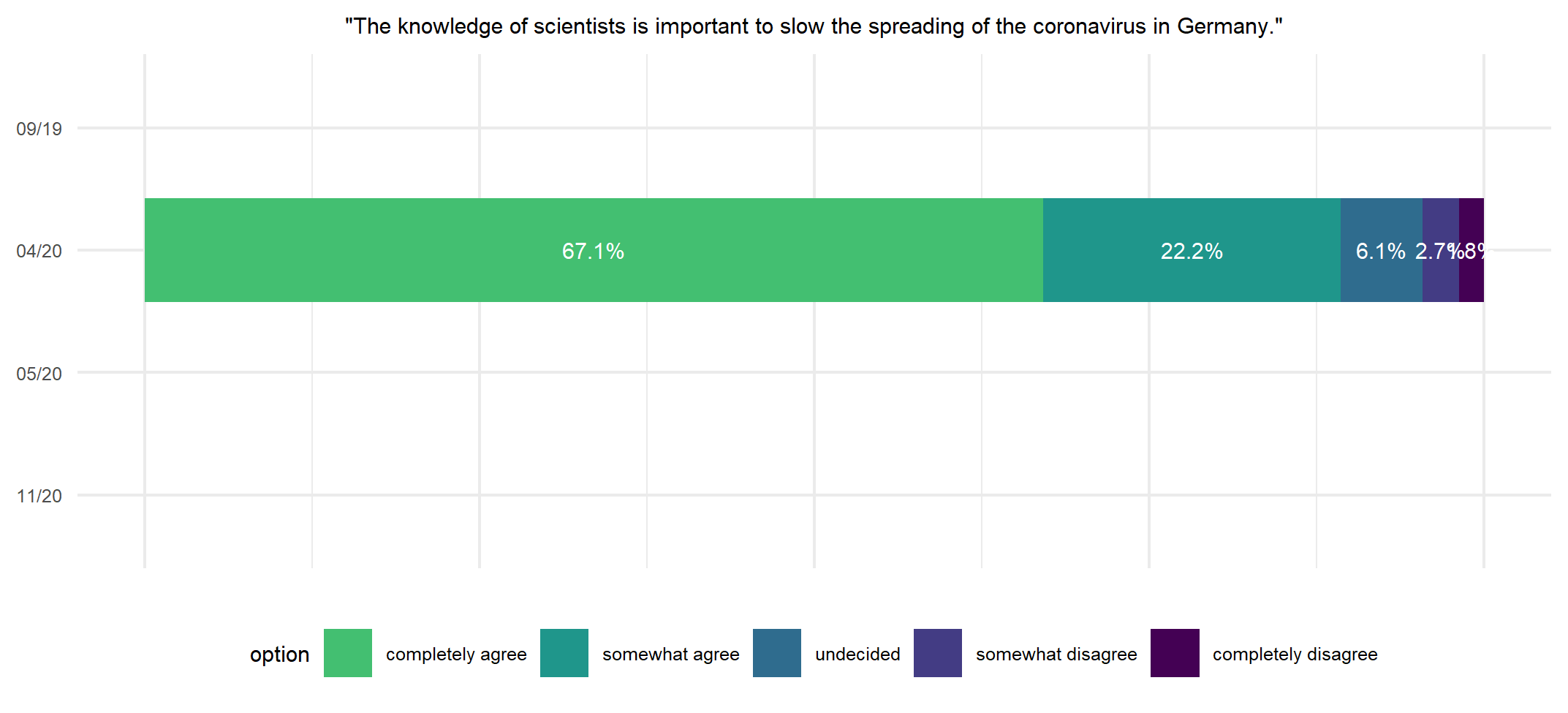

Supplement: S17 Fig — (TIFF) [file pone.0262823.s017.tiff]

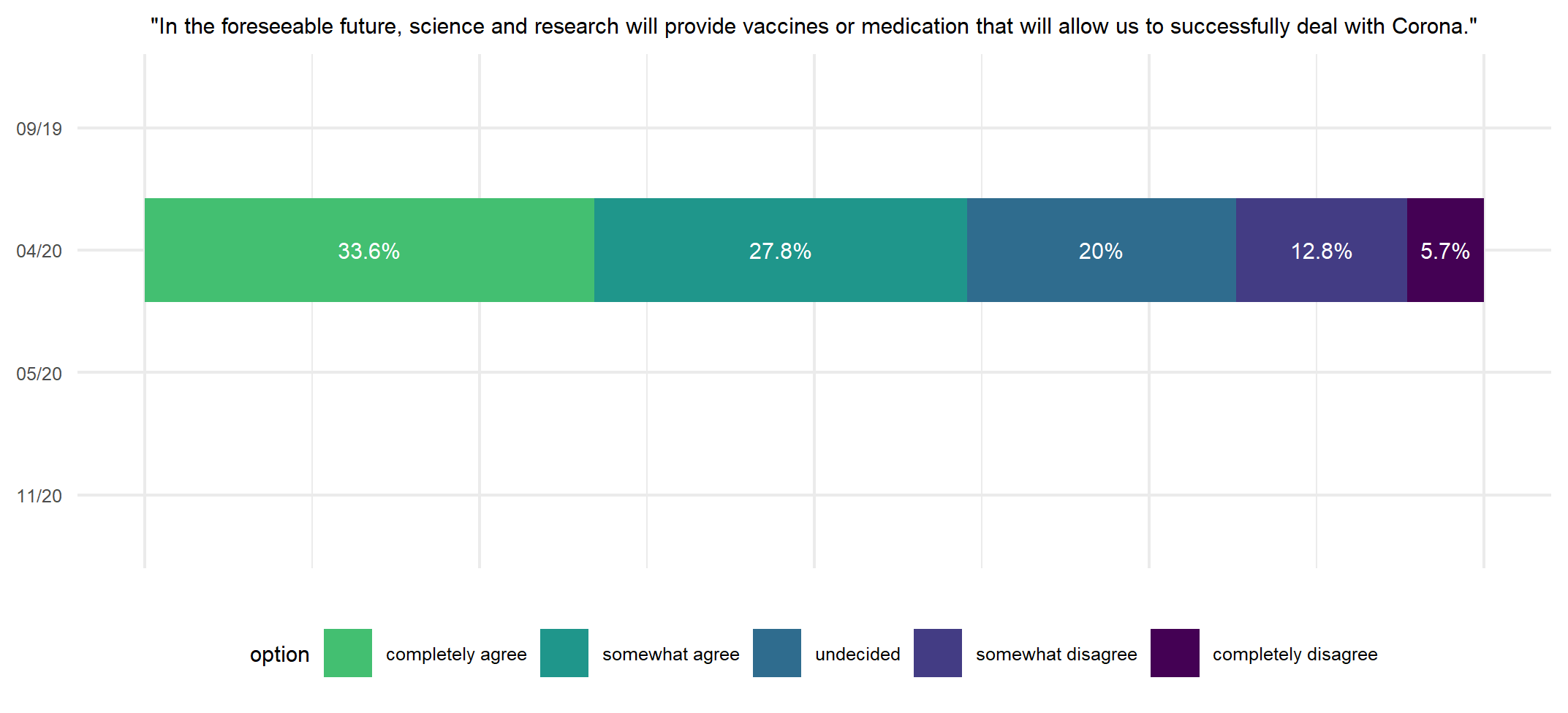

Supplement: S18 Fig — (TIFF) [file pone.0262823.s018.tiff]

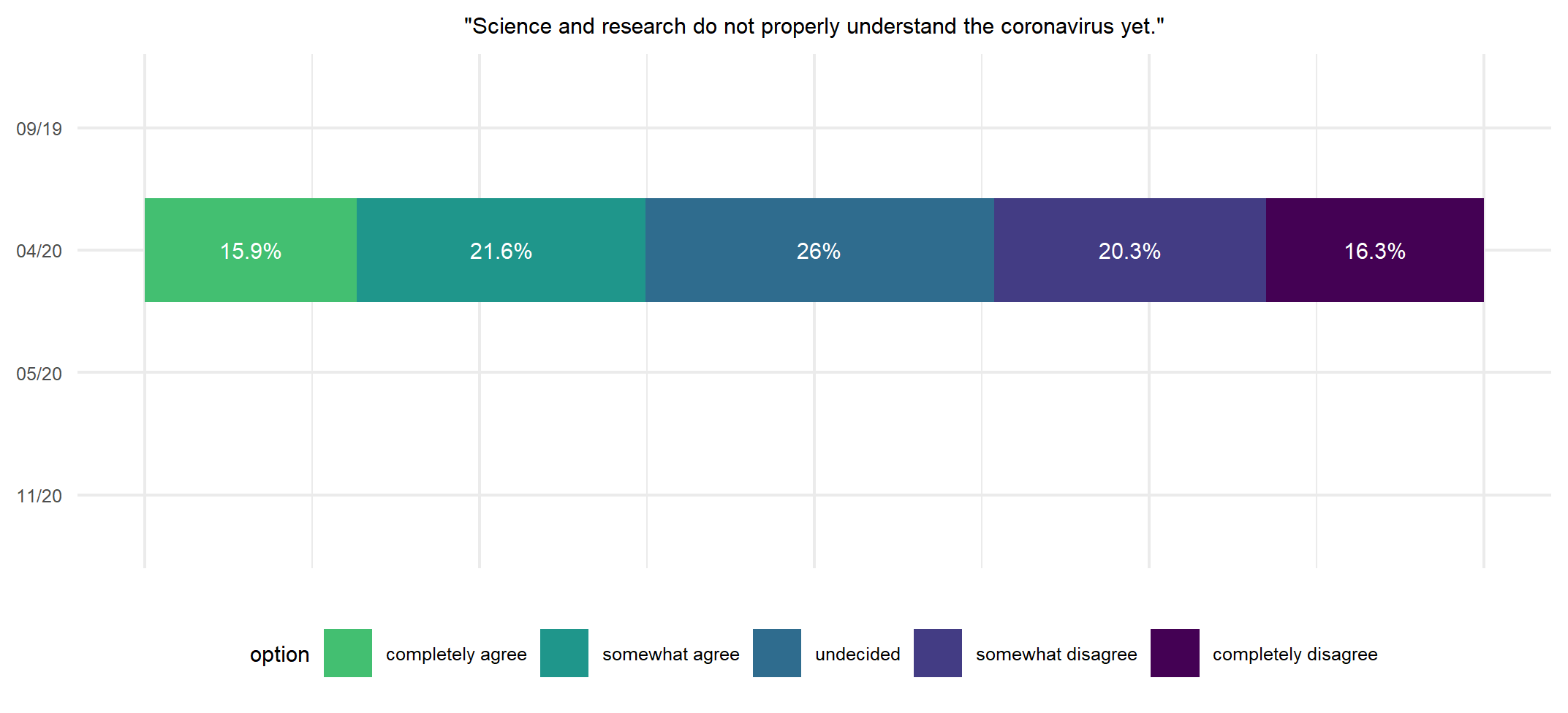

Supplement: S19 Fig — (TIFF) [file pone.0262823.s019.tiff]

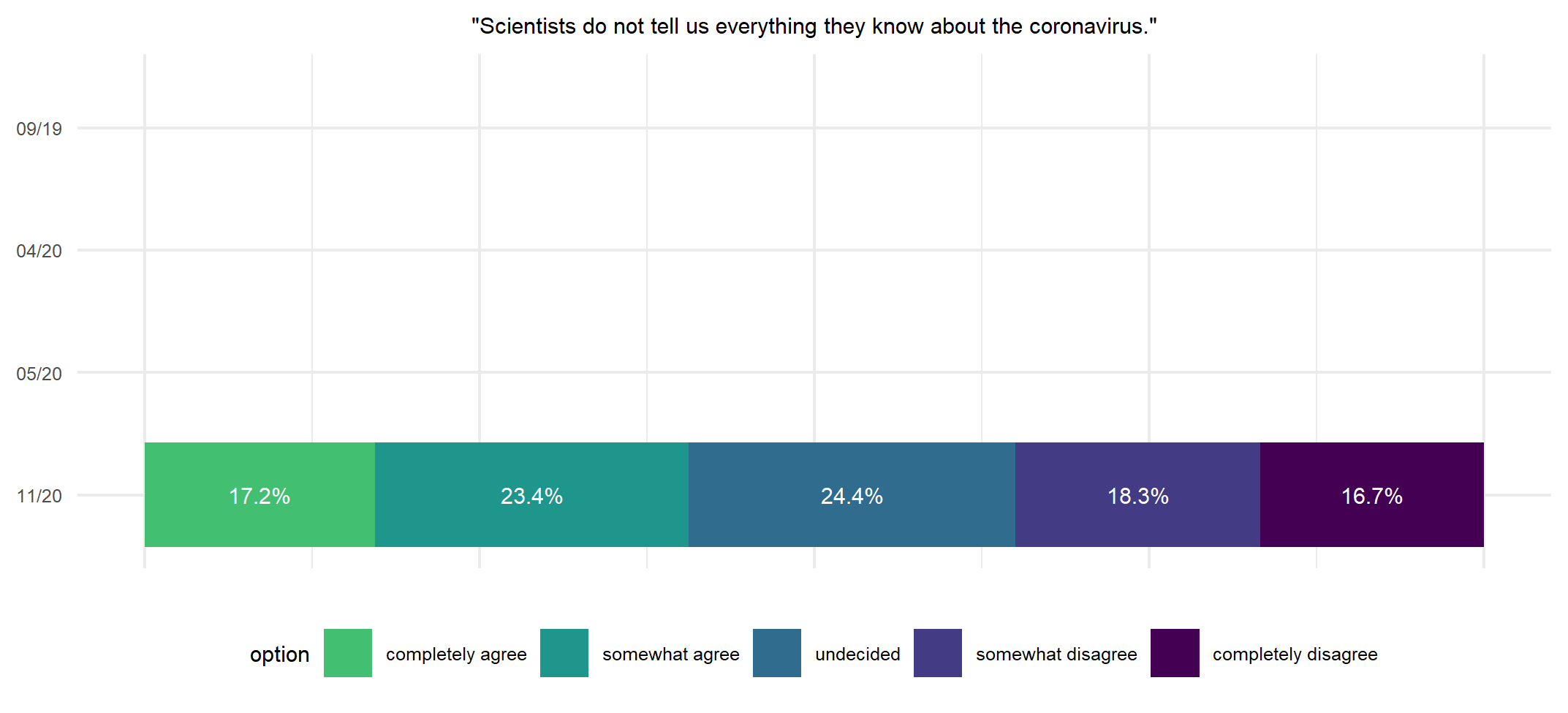

Supplement: S20 Fig — (TIFF) [file pone.0262823.s020.tiff]

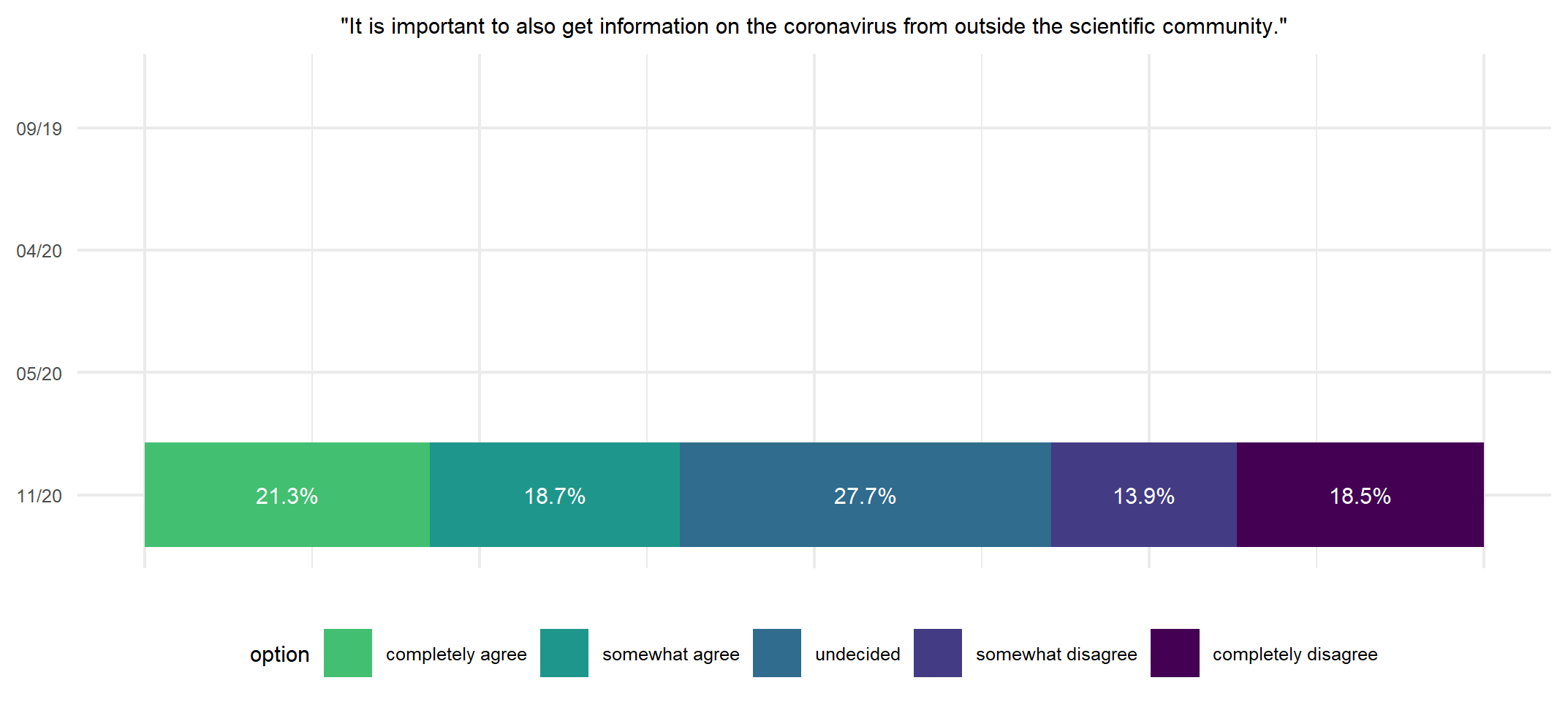

Supplement: S21 Fig — (TIFF) [file pone.0262823.s021.tiff]

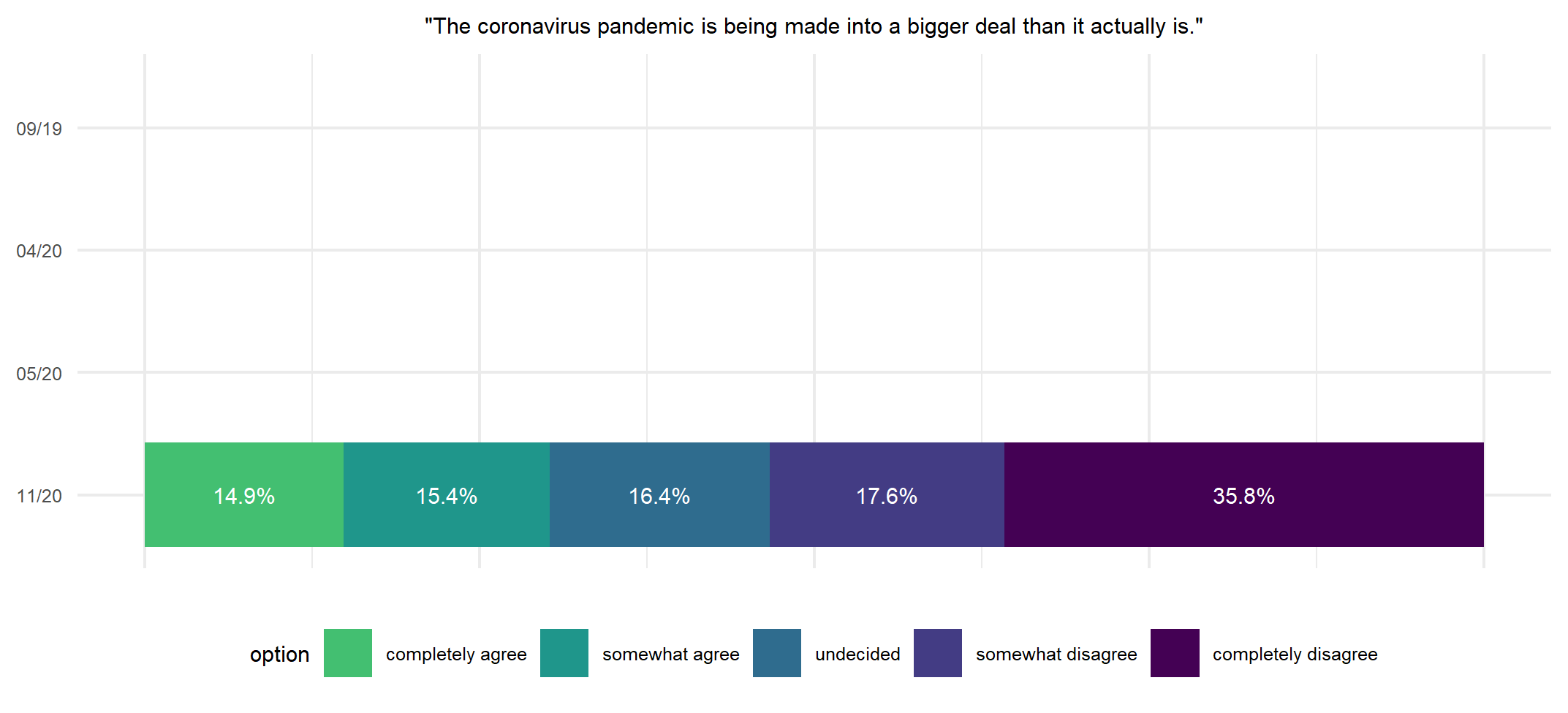

Supplement: S22 Fig — (TIFF) [file pone.0262823.s022.tiff]

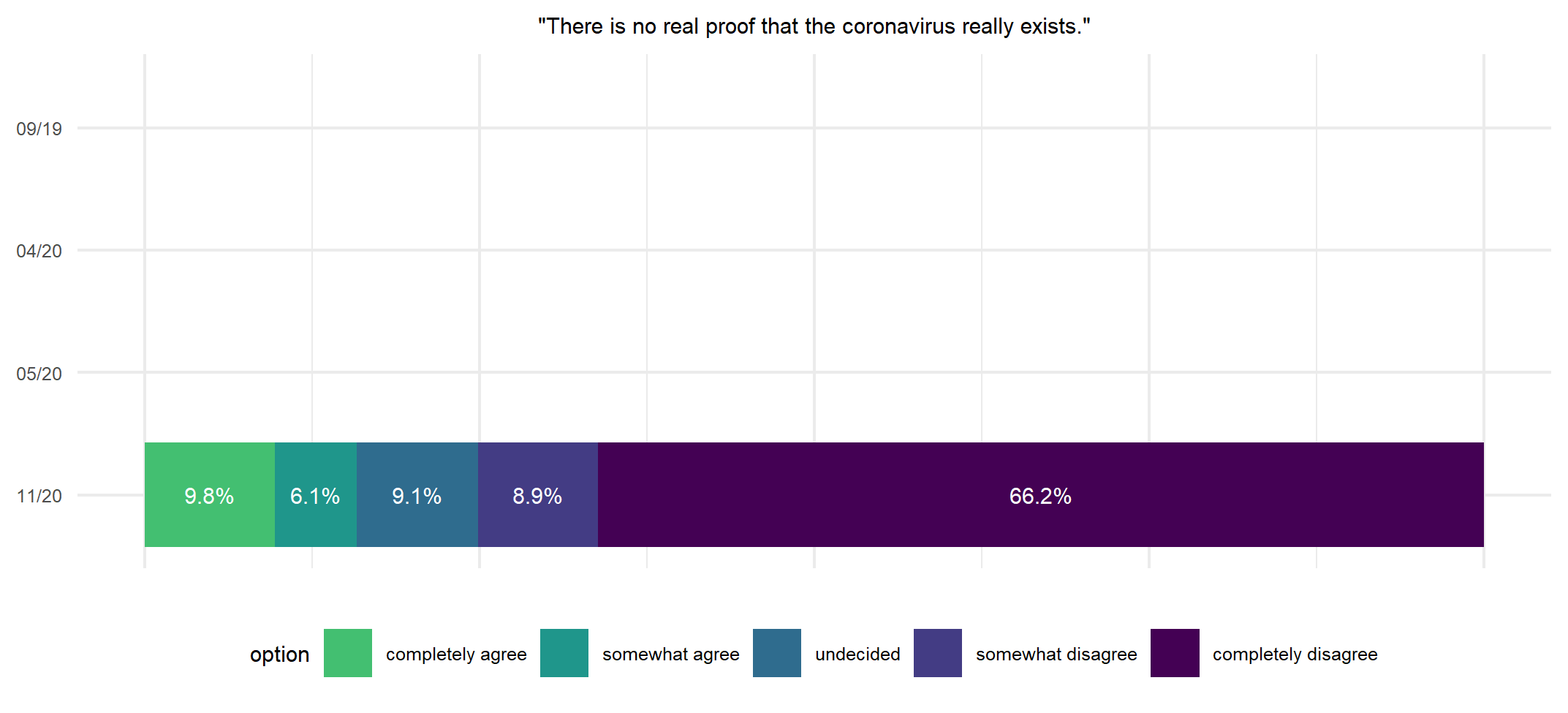

Supplement: S23 Fig — (TIFF) [file pone.0262823.s023.tiff]

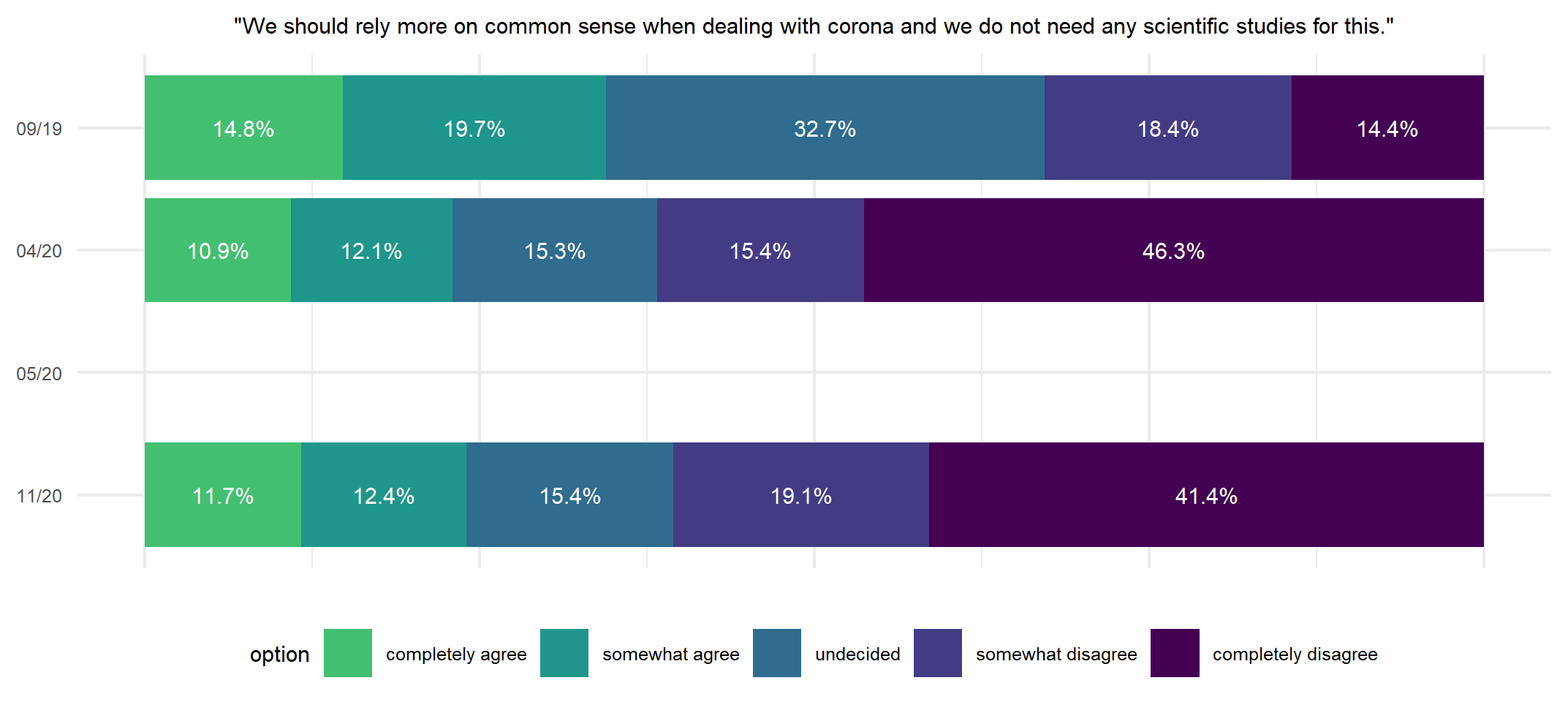

Supplement: S24 Fig — (TIFF) [file pone.0262823.s024.tiff]

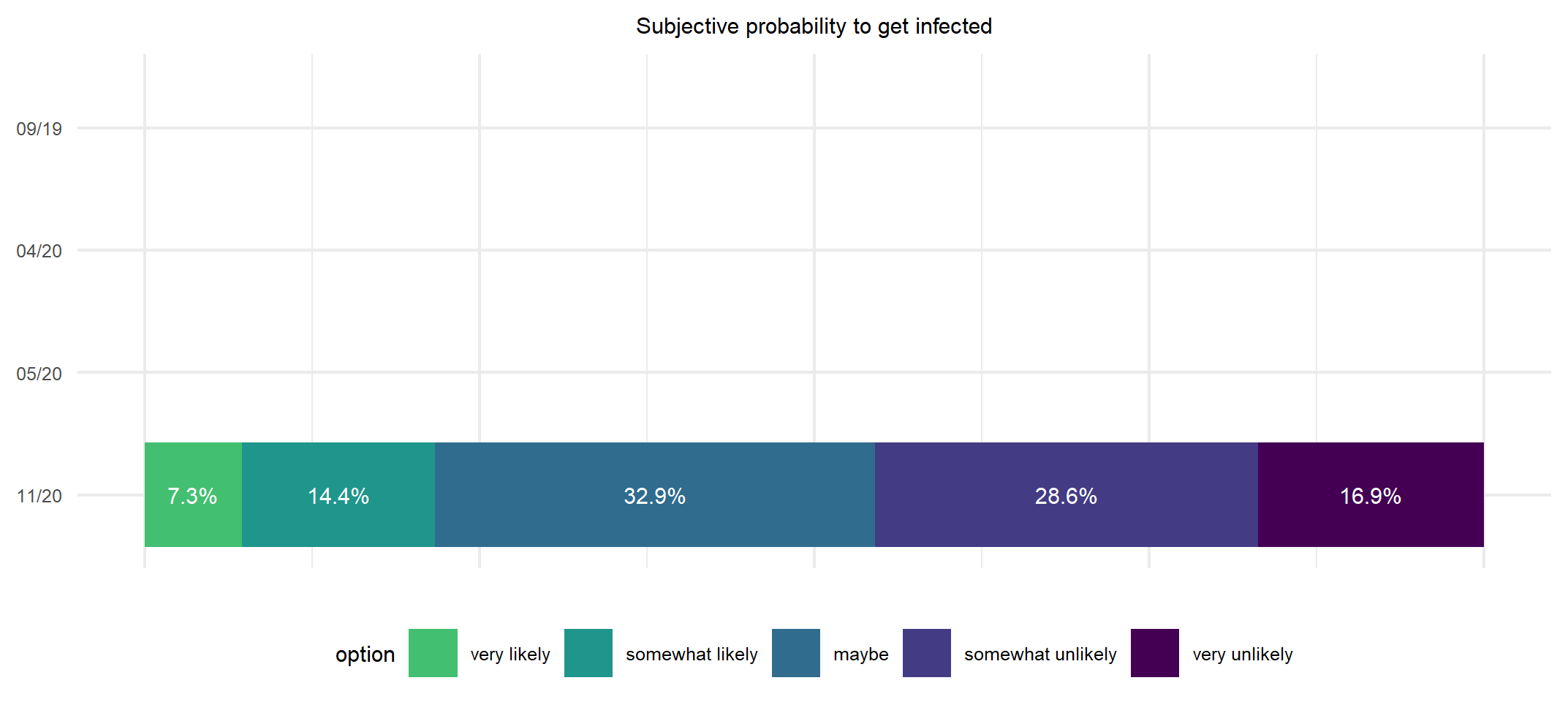

Supplement: S25 Fig — (TIFF) [file pone.0262823.s025.tiff]

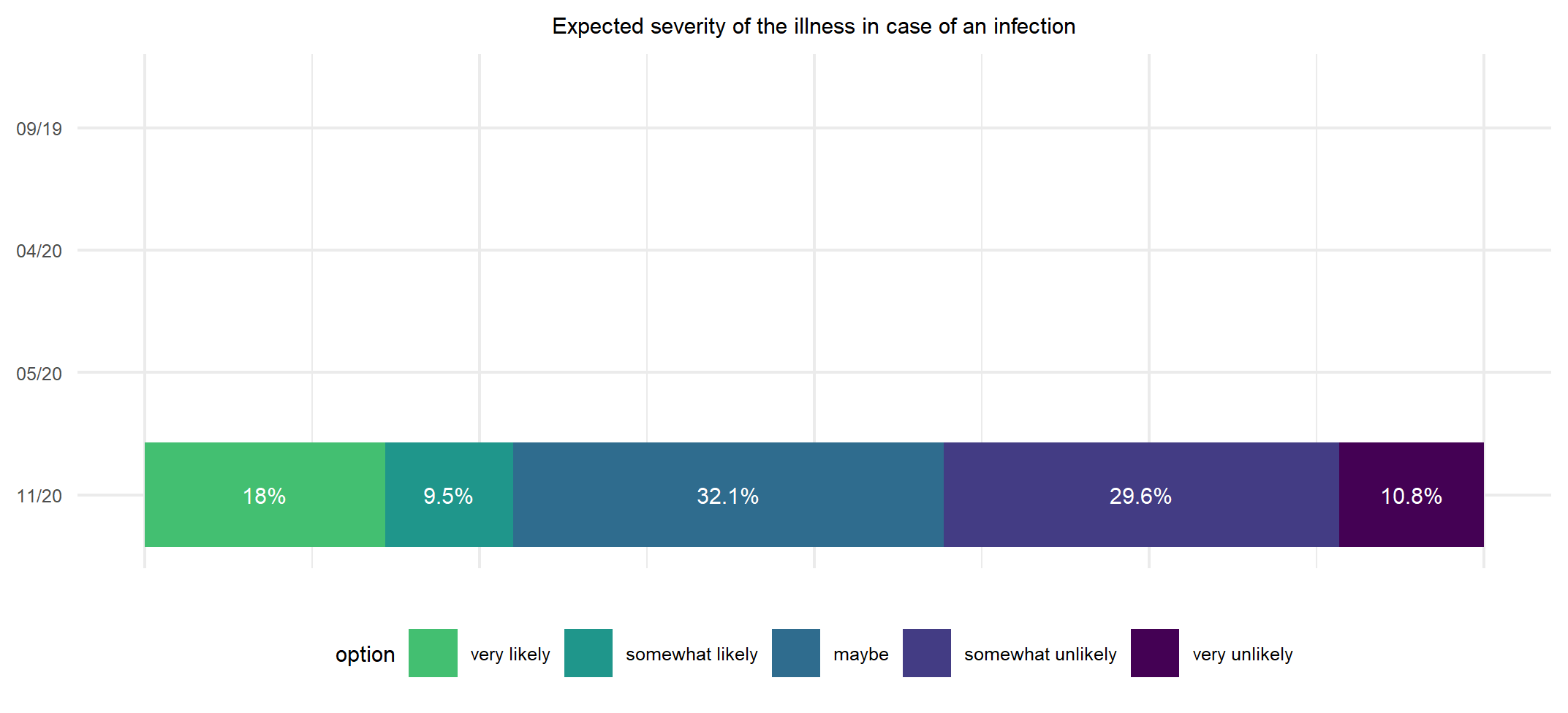

Supplement: S26 Fig — (TIFF) [file pone.0262823.s026.tiff]

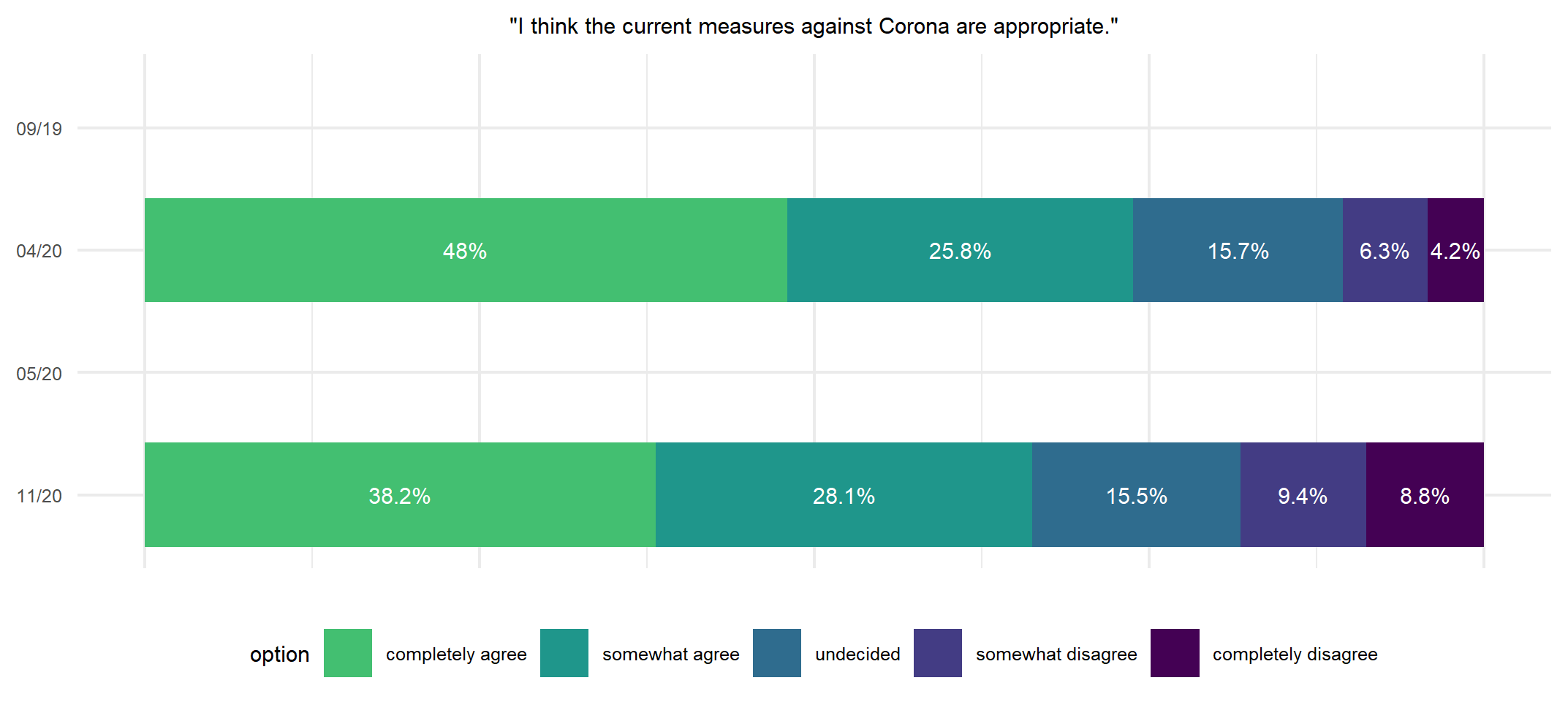

Supplement: S27 Fig — (TIFF) [file pone.0262823.s027.tiff]

*
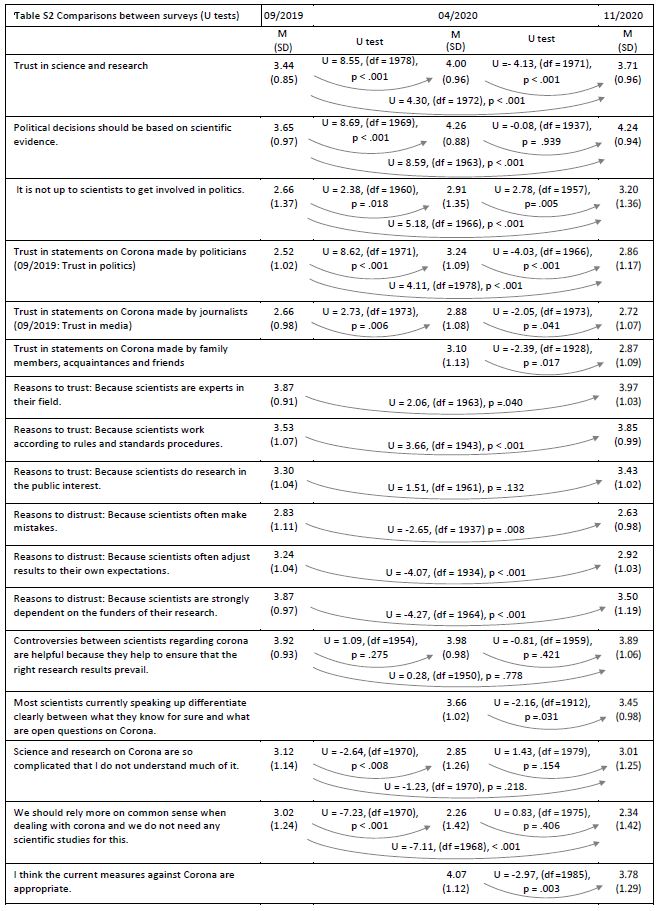
*

Supplement: S2 Table — (DOCX) [file pone.0262823.s029.docx]
