# Supplementary material for: An anchor in troubled times: Trust in science before and within the COVID-19 pandemic
Source: PLoS One. 2022 Feb 9;17(2):e0262823. doi: 10.1371/journal.pone.0262823 (PMC8827432; doi:10.1371/journal.pone.0262823)
Supplement: S1 Table — (DOCX) [file pone.0262823.s028.docx]

| **Table S1**. Descriptive statistics for trust in science and research in subgroups | | | | | | | | |
| --- | --- | --- | --- | --- | --- | --- | --- | --- |
|  | Trust in science and research | | | | | | | |
|  | 09/2019 | | 04/2020 | | 05/2020 | | 11/2020 | |
| Subgroup | *M* | (*SD*) | *M* | (*SD*) | *M* | (*SD*) | *M* | (*SD*) |
| Full sample | 3.44 | (0.85) | 4.00 | (0.96) | 3.87 | (0.99) | 3.71 | (0.96) |
| Gender: male | 3.58 | (0.86) | 3.99 | (0.97) | 3.95 | (0.99) | 3.79 | (0.98) |
| Gender: female | 3.30 | (0.81) | 4.01 | (0.94) | 3.80 | (0.98) | 3.62 | (0.93) |
| Age: younger than 60 years | 3.50 | (0.84) | 4.03 | (0.98) | 3.93 | (0.96) | 3.71 | (0.98) |
| Age: 60 years or older | 3.30 | (0.87) | 3.94 | (0.91) | 3.75 | (1.04) | 3.69 | (0.92) |
| Education: Lower than A-level/no degree | 3.32 | (0.83) | 3.86 | (0.96) | 3.69 | (1.01) | 3.48 | (0.95) |
| Education: A-level or higher | 3.69 | (0.85) | 4.28 | (0.88) | 4.23 | (0.84) | 4.14 | (0.82) |
| Children aged < 14 years in household: no | 3.41 | (0.87) | 4.04 | (0.97) | 3.89 | (0.98) | 3.74 | (0.96) |
| Children aged < 14 years in household: yes | 3.58 | (0.67) | 3.86 | (0.87) | 3.80 | (1.02) | 3.58 | (0.96) |
| Populist party preference: other/no preference/don't know | 3.43 | (0.85) | 4.05 | (0.94) | 3.89 | (1.00) | 3.75 | (0.94) |
| Populist party preference: AfD | 3.45 | (0.66) | 3.38 | (1.11) | 3.81 | (1.25) | 3.04 | (0.81) |

*Note.* Descriptive statistics were computed using survey weights (R package survey v4.0; Lumley, 2020).
