# Supplementary material for: An anchor in troubled times: Trust in science before and within the COVID-19 pandemic
Source: PLoS One. 2022 Feb 9;17(2):e0262823. doi: 10.1371/journal.pone.0262823 (PMC8827432; doi:10.1371/journal.pone.0262823)
Supplement: S4 Table — (DOCX) [file pone.0262823.s031.docx]

| **Table S4.** Predicting changes in trust in science and research before and during the Covid-19 pandemic | | | | | | | | | | | | | | | | |
| --- | --- | --- | --- | --- | --- | --- | --- | --- | --- | --- | --- | --- | --- | --- | --- | --- |
|  | Trust in science and research | | | | | | | | | | | | | | | |
|  | 09/2019 vs. 04/2020 | | | | 09/2019 vs. 11/2020 | | | | 04/2020 vs. 05/2020 | | | | 04/2020 vs. 11/2020 | | | |
|  | *b* | *p* | 95% CI | *SE* | *b* | *p* | 95% CI | *SE* | *b* | *p* | 95% CI | *SE* | *b* | *p* | 95% CI | *SE* |
| Intercept | **2.86** | **<.001** | **[2.48, 3.20]** | .18 | **2.86** | **<.001** | **[2.49, 3.20]** | 0.18 | **2.63** | **<.001** | [1.92, 3.27] | 0.34 | **2.63** | **<.001** | **[1.95, 3.29]** | 0.34 |
| Time | -0.22 | .568 | [-1.06, 0.44] | .39 | -0.42 | .136 | [-0.94, 0.18] | 0.28 | 0.01 | .975 | [-0.77, 0.88] | 0.42 | -0.20 | .622 | [-1.02, 0.58] | 0.41 |
| Gender (1 = female) | -0.16 | .062 | [-0.33, 0.01] | .09 | -0.16 | .062 | [-0.34, 0.00] | 0.09 | -0.06 | .556 | [-0.26, 0.16] | 0.11 | -0.06 | .556 | [-0.26, 0.15] | 0.11 |
| Age (1 = 60 years or older) | -0.11 | .258 | [-0.29, 0.09] | .10 | -0.11 | .258 | [-0.30, 0.08] | 0.10 | -0.15 | .228 | [-0.38, 0.10] | 0.12 | -0.15 | .228 | [-0.38, 0.09] | 0.12 |
| Education (1 = A-level) | **0.24** | **.008** | **[0.06, 0.41]** | .09 | **0.24** | **.008** | [0.07, 0.42] | 0.09 | **0.29** | **.009** | **[0.06, 0.49]** | 0.11 | **0.29** | **.009** | **[0.06, 0.50]** | 0.11 |
| Children aged < 14 years in household (1 = yes) | 0.00 | .968 | [-0.19, 0.23] | .11 | 0.00 | .968 | [-0.19, 0.22] | 0.11 | -0.17 | .219 | [-0.45, 0.08] | 0.14 | -0.17 | .219 | [-0.47, 0.07] | 0.14 |
| Populist party preference  (1 = AfD) | 0.11 | .352 | [-0.13, 0.35] | .12 | 0.11 | .352 | [-0.13, 0.36] | 0.12 | -0.57 | .057 | [-1.14, 0.01] | 0.30 | -0.57 | .057 | [-1.18, 0.04] | 0.30 |
| Political decisions should be based on scientific evidence.^a^ | **0.17** | **<.001** | **[0.08, 0.26]** | .05 | **0.17** | **<.001** | [0.08, 0.26] | 0.05 | **0.34** | **<.001** | **[0.20, 0.48]** | 0.07 | **0.34** | **<.001** | **[0.21, 0.48]** | 0.07 |
| Time x gender | 0.10 | .476 | [-0.18, 0.37] | .14 | -0.01 | .945 | [-0.27, 0.24] | 0.12 | -0.14 | .364 | [-0.45, 0.15] | 0.15 | -0.11 | .446 | [-0.42, 0.15] | 0.14 |
| Time x age | -0.04 | .810 | [-0.35, 0.26] | .16 | 0.09 | .502 | [-0.18, 0.35] | 0.13 | 0.01 | .931 | [-0.31, 0.33] | 0.16 | 0.13 | .405 | [-0.18, 0.43] | 0.15 |
| Time x education | 0.05 | .715 | [-0.24, 0.32] | .14 | **0.32** | **.013** | **[0.06, 0.57]** | **0.13** | -0.04 | .814 | [-0.35, 0.25] | 0.15 | 0.26 | .065 | [-0.01, 0.56] | 0.14 |
| Time x children aged < 14 years | -0.17 | .318 | [-0.52, 0.14] | .17 | -0.22 | .215 | [-0.60, 0.11] | 0.18 | 0.15 | .545 | [-0.25, 0.69] | 0.25 | -0.05 | .804 | [-0.45, 0.32] | 0.20 |
| Time x populist party preference | **-0.68** | **.034** | **[-1.32, -0.02]** | .32 | **-0.58** | **.027** | **[-1.08, -0.05]** | **0.26** | 0.23 | .611 | [-0.76, 1.03] | 0.45 | 0.10 | .786 | [-0.65, 0.88] | 0.38 |
| Time x political decisions […]^a^ | **0.17** | **.037** | **[0.02, 0.35]** | .08 | 0.13 | .061 | [-0.01, 0.25] | 0.07 | -0.01 | .899 | [-0.19, 0.15] | 0.09 | -0.04 | .609 | [-0.21, 0.12] | 0.08 |
| *Adj. R²* | .21 | | | | .19 | | | | .19 | | | | .21 | | | |
| *F value* | *F*(13, 1814) = 15.39, *p* < .001 | | | | *F*(13, 1816) = 18.43, *p* < .001 | | | | *F*(13, 1863) = 10.99, *p* < .001 | | | | *F*(13, 1843) = 18.19, *p* < .001 | | | |
| *N* | 1829 | | | | 1831 | | | | 1878 | | | | 1858 | | | |

*Note*. Analyses used survey weights and were computed using the R package survey v4.0 (Lumley, 2020). In all regression models, the assumption of normality of the residuals was violated (which can be retraced by running the R syntax we share, see Methods section); therefore, standard errors and confidence interval bounds (95%, two-sided) of *b* coefficients were bootstrapped. Bootstrapping was done with the R package boot v1.3-25 (Ripley, 2020) using the bias-corrected and accelerated method (BC_a_; DiCiccio & Efron, 1996), which accounts for the skewness and lack of symmetry in the observed data (Carpenter & Bithell, 2000). Boldface = p < .05.

^a^ In the 09/2019 wave, this item was introduced as referring to climate change research and policy-making; in the 04/2020, 05/2020 and 11/2020 waves it was introduced as referring to the Covid19 pandemic.
