# Supplementary material for: An anchor in troubled times: Trust in science before and within the COVID-19 pandemic
Source: PLoS One. 2022 Feb 9;17(2):e0262823. doi: 10.1371/journal.pone.0262823 (PMC8827432; doi:10.1371/journal.pone.0262823)
Supplement: S5 Table — (DOCX) [file pone.0262823.s032.docx]

| **Table S5.** Predicting changes in *trust in science* *and research* before and during the Covid-19 pandemic focusing on beliefs in science measured in 04/2020 and 11/2020) | | | | |
| --- | --- | --- | --- | --- |
|  | Trust in science and research | | | |
|  | 04/2020 vs. 11/2020 | | | |
|  | *b* | *p* | 95% CI | *SE* |
| Intercept | **2.20** | **<.001** | **[1.24, 3.20]** | 0.49 |
| Time (1 = 11/2020) | 0.30 | .621 | [-0.94, 1.48] | 0.60 |
| Gender (1 = female) | -0.10 | .300 | [-0.27, 0.09] | 0.09 |
| Age (1 = 60 years or older) | -0.19 | .087 | [-0.41, 0.01] | 0.11 |
| Education (1 = A-level) | 0.14 | .135 | [-0.05, 0.32] | 0.09 |
| Children aged < 14 years in household (1 = yes) | -0.15 | .245 | [-0.41, 0.09] | 0.13 |
| Populist party preference (1 = AfD) | -0.27 | .397 | [-0.92, 0.36] | 0.32 |
| Political decisions should be based on scientific evidence. ^a^ | **0.13** | **.048** | **[-0.02, 0.26]** | 0.06 |
| It is not up to scientists to get involved in politics. | -0.01 | .799 | [-0.08, 0.06] | 0.04 |
| Trust in statements on Corona made by politicians (09/2019: Trust in politics) | 0.11 | .101 | [-0.02, 0.26] | 0.07 |
| Trust in statements on Corona made by journalists (09/2019: Trust in media) | **0.22** | **<.001** | **[0.11, 0.36]** | 0.06 |
| Trust in statements on Corona made by family members, acquaintances and friends | -0.06 | .328 | [-0.18, 0.06] | 0.06 |
| Controversies between scientists regarding Corona are helpful because they help to ensure that the right research results prevail. | **0.22** | **<.001** | **[0.10, 0.34]** | 0.06 |
| Most scientists currently speaking up differentiate clearly between what they know for sure and what are open questions on Corona. | 0.05 | .382 | [-0.15, 0.06] | 0.05 |
| Science and research on Corona are so complicated that I do not understand much of it. | -0.05 | .238 | [-0.13, 0.03] | 0.04 |
| We should rely more on common sense when dealing with Corona and we do not need any scientific studies for this. | **-0.10** | **.034** | **[-0.18, -0.01]** | 0.05 |
| I think the current measures against Corona are appropriate. | 0.06 | .234 | [-0.03, 0.16] | 0.05 |
| Time x gender | -0.15 | .250 | [-0.43, 0.08] | 0.13 |
| Time x age | 0.11 | .434 | [-0.17, 0.41] | 0.14 |
| Time x education | 0.20 | .100 | [-0.05, 0.45] | 0.12 |
| Time x children ages < 14 years | -0.00 | .999 | [-0.35, 0.33] | 0.18 |
| Time x Populist party preference | 0.16 | .697 | [-0.64, 1.00] | 0.41 |
| Time x political decisions […]^a^ | -0.02 | .858 | [-0.17, 0.17] | 0.09 |
| Time x it is not up to scientists to get involved in politics | 0.04 | .460 | [-0.06, 0.14] | 0.05 |
| Time x trust in statements on Corona made by politicians […] | -0.04 | .648 | [-0.23, 0.13] | 0.09 |
| Time x trust in statements on Corona made by journalists […] | -0.05 | .561 | [-0.23, 0.10] | 0.08 |
| Time x trust in statements on Corona made by family members […] | 0.01 | .888 | [-0.14, 0.17] | 0.08 |
| Time x controversies between scientists regarding Corona are helpful […] | 0.09 | .230 | [-0.25, 0.06] | 0.08 |
| Time x most scientists currently speaking up differentiate clearly […] | 0.04 | .614 | [-0.12, 0.16] | 0.07 |
| Time x science and research on Corona are so complicated […] | -0.05 | .400 | [-0.16, 0.07] | 0.05 |
| Time x we should rely more on common sense […] | 0.02 | .748 | [-0.11, 0.14] | 0.06 |
| Time x I think the current measures […] | -0.01 | .883 | [-0.13, 0.12] | 0.07 |
| *Adj. R²* | .35 | | | |
| *F value* | *F*(31, 1664) = 14.54, *p* < .001 | | | |
| *N* | 1697 | | | |

*Note*. Analyses used survey weights and were computed using the R package survey v4.0 (Lumley, 2020). In all regression models, the assumption of normality of the residuals was violated (which can be retraced by running the R syntax we share, see Methods section) ; therefore, standard errors and confidence interval bounds (95%, two-sided) of *b* coefficients were bootstrapped. Bootstrapping was done with the R package boot v1.3-25 (Ripley, 2020) using the bias-corrected and accelerated method (BC_a_; DiCiccio & Efron, 1996), which accounts for the skewness and lack of symmetry in the observed data (Carpenter & Bithell, 2000). Boldface = p < .05.

^a^ In the 09/2019 wave, this item was introduced as referring to climate change research and policy-making; in the 04/2020, 05/2020 and 11/2020 waves it was introduced as referring to the Covid19 pandemic.
