# Supplementary material for: An anchor in troubled times: Trust in science before and within the COVID-19 pandemic
Source: PLoS One. 2022 Feb 9;17(2):e0262823. doi: 10.1371/journal.pone.0262823 (PMC8827432; doi:10.1371/journal.pone.0262823)
Supplement: S6 Table — (DOCX) [file pone.0262823.s033.docx]

| **Table S6.** Predicting trust in science with additionally focusing beliefs in science (04/2020), respectively skeptical beliefs and trust/distrust reasons (11/2020) | | | | | | | | |
| --- | --- | --- | --- | --- | --- | --- | --- | --- |
|  | Trust in science and research | | | | | | | |
|  | 04/2020 | | | | 11/2020 | | | |
|  | *b* | *p* | 95% CI | *SE* | *b* | *p* | 95% CI | *SE* |
| Intercept | **2.13** | **<.001** | **[1.18, 3.15]** | 0.50 | **1.76** | **<.001** | **[0.96, 2.64]** | 0.41 |
| Gender (1 = female) | -0.09 | .320 | [-0.26, 0.11] | 0.09 | **-0.19** | **.010** | **[-0.34, -0.04]** | 0.07 |
| Age (1 = 60 years or older) | -0.19 | .090 | [-0.41, 0.04] | 0.11 | 0.00 | .991 | [-0.16, 0.23] | 0.09 |
| Education (1 = A-level) | 0.14 | .142 | [-0.05, 0.32] | 0.09 | **0.33** | **<.001** | **[0.18, 0.47]** | 0.07 |
| Children aged < 14 years in household (1 = yes) | -0.15 | .245 | [-0.41, 0.09] | 0.13 | -0.13 | .218 | [-0.33, 0.09] | 0.10 |
| Populist party preference (1 = AfD) | -0.26 | .408 | [-0.90, 0.36] | 0.32 | -0.02 | .934 | [-0.50, 0.57] | 0.25 |
| Political decisions should be based on scientific evidence. | 0.12 | .070 | [-0.02, 0.24] | 0.07 | 0.10 | .072 | [-0.02, 0.20] | 0.05 |
| It is not up to scientists to get involved in politics. | -0.01 | .795 | [-0.08, 0.07] | 0.04 | 0.04 | .125 | [-0.01, 0.10] | 0.03 |
| Trust in statements on Corona made by politicians (09/2019: Trust in politics) | 0.11 | .105 | [-0.02, 0.25] | 0.07 | 0.05 | .360 | [-0.06, 0.15] | 0.05 |
| Trust in statements on Corona made by journalists (09/2019: Trust in media) | **0.22** | **<.001** | **[0.11, 0.34]** | 0.06 | **0.15** | **.002** | **[0.06, 0.26]** | 0.05 |
| Trust in statements on Corona made by family members, acquaintances and friends | -0.06 | .326 | [-0.19, 0.05] | 0.06 | -0.02 | .576 | [-0.11, 0.06] | 0.04 |
| Controversies between scientists regarding Corona are helpful because they help to ensure that the right research results prevail. | **0.22** | **<.001** | **[0.11, 0.34]** | 0.06 | 0.07 | .078 | [-0.01, 0.17] | 0.04 |
| Most scientists currently speaking up differentiate clearly between what they know for sure and what are open questions on Corona. | -0.05 | .373 | [-0.16, 0.05] | 0.05 | -0.01 | .875 | [-0.09, 0.07] | 0.04 |
| Science and research on Corona are so complicated that I do not understand much of it. | -0.05 | .254 | [-0.14, 0.03] | 0.04 | **-0.07** | **.042** | **[-0.14, 0.00]** | 0.03 |
| We should rely more on common sense when dealing with Corona and we do not need any scientific studies for this. | **-0.10** | **.033** | **[-0.18, -0.01]** | 0.05 | 0.03 | .595 | [-0.06, 0.13] | 0.05 |
| I think the current measures against Corona are appropriate. | 0.06 | .261 | [-0.04, 0.16] | 0.05 | -0.00 | .989 | [-0.07, 0.09] | 0.04 |
| Beliefs in the promise of science and research on Covid ^a^ | 0.03 | .689 | [-0.12, 0.17] | 0.07 |  |  |  |  |
| Skeptical beliefs regarding the pandemic ^b^ |  |  |  |  | **-0.20** | **.010** | **[-0.40, -0.06]** | 0.08 |
| Reasons to trust: Because scientists are experts in their field. |  |  |  |  | **0.13** | **.018** | **[0.03, 0.25]** | 0.06 |
| Reasons to trust: Because scientists work according to rules and standards procedures |  |  |  |  | **0.13** | **.004** | **[0.03, 0.22]** | 0.05 |
| Reasons to trust: Because scientists do research in the public interest. |  |  |  |  | 0.02 | .584 | [-0.07, 0.10] | 0.04 |
| Reasons to distrust: Because scientists often make mistakes. |  |  |  |  | 0.08 | .140 | [-0.01, 0.19] | 0.05 |
| Reasons to distrust: Because scientists often adjust results to their own expectations. |  |  |  |  | 0.02 | .735 | [-0.09, 0.10] | 0.05 |
| Reasons to distrust: Because scientists are strongly dependent on the funders of their research. |  |  |  |  | -0.03 | .437 | [-0.12, 0.06] | 0.04 |
| Subjective probability to get infected and expected severity in case of infection ^c^ |  |  |  |  | 0.03 | .605 | [-0.10, 0.13] | 0.06 |
| *Adj. R²* | .33 | | | | .42 | | | |
| *F value* | *F*(16, 815) = 12.85, *p* < .001 | | | | *F*(23, 791) = 15.34, *p* < .001 | | | |
| *N* | 832 | | | | 815 | | | |

*Note.* Analyses used survey weights and were computed using the R package survey v4.0 (Lumley, 2020). In all regression models, the assumption of normality of the residuals was violated (which can be retraced by running the R syntax we share, see Methods section); therefore, standard errors and confidence interval bounds (95%, two-sided) of *b* coefficients were bootstrapped. Bootstrapping was done with the R package boot v1.3-25 (Ripley, 2020) using the bias-corrected and accelerated method (BC_a_; DiCiccio & Efron, 1996), which accounts for the skewness and lack of symmetry in the observed data (Carpenter & Bithell, 2000). Boldface = p < .05.

^a^ Beliefs in the promise of science and research on Covid represents the mean value across the three survey items “The knowledge of scientists is important to slow the spreading of the Coronavirus in Germany.”, “In the foreseeable future, science and research will provide vaccines or medication that will allow us to successfully deal with Corona.”, “Science and research do not properly understand the Coronavirus yet.”

^b^ Skeptikal beliefs regarding the pandemic = mean score of survey items represents the mean value across the four survey items “Scientists do not tell us everything they know about the Coronavirus.”, ”It is important to also get information on the Coronavirus from outside the scientific community.”, “The Coronavirus pandemic is being made into a bigger deal than it actually is.”, “There is no real proof that the Coronavirus really exists.”

^c^ Subjective probability to get infected and expected severity in case of infection = mean score of subjective probability to get infected and expected severity of the illness in case of an infection.
